# Strengthening of corroded RC slab–column joints using thin-ply hybrid FRP under punching shear

**DOI:** 10.1038/s41598-026-36610-2

**Published:** 2026-02-14

**Authors:** Ahmed M. Gomaa, Manar A. Ahmed, Sherif A. Khafaga, Ehab M. Lotfy, Sally Hosny

**Affiliations:** 1 Department of Construction and Building Engineering, Faculty of Engineering and Technology, Egyptian Chinese University, Cairo, Egypt; 2 Department of Civil Engineering, Faculty of Engineering, Suez Canal University, Ismailia, Egypt; 3 Faculty of Engineering, Taibah University, Madina, Saudi Arabia; 4 Building Materials Research and Quality Control Institute, Housing and Building National Research Center (HBRC), Cairo, Egypt

**Keywords:** Slab-column joint, CFRP, GFRP, Hybrid Composites, Corrosion acceleration, Punching shear capacity, Engineering, Materials science

## Abstract

A comprehensive investigation into the punching shear (PS) strengthening behavior of corroded two-way reinforced concrete (RC) slabs using thin-ply glass/carbon hybrid FRP was conducted through an integrated experimental, numerical, and theoretical program. Eleven slab–column joints (SCJs) were cast and tested under monotonic loading to assess different external strengthening configurations. The experimental results indicated that hybrid thin-ply FRP improved PS capacity more effectively than single-material GFRP or CFRP systems within the tested conditions. Numerical analysis using ABAQUS was then carried out to evaluate the influence of strengthening parameters. The simulations showed that increasing the FRP layers beyond two, or thickness beyond approximately 0.6 mm, resulted in limited additional capacity due to premature debonding. The parametric study identified a skewed strip layout with a 50 mm offset from the column face and two hybrid layers (0.6 mm each) as the most effective configuration for the investigated geometry and corrosion level. Strengthening efficiency was observed to decrease as corrosion severity increased, reducing from 50.77% at 5% corrosion to 24.76% at 30%. Overall, the findings suggest that thin-ply hybrid FRP, when optimally arranged, can be a practical option for partial recovery of PS capacity in moderately corroded RC slab–column joints; however, its effectiveness is influenced by corrosion extent, bond performance, and configuration, and therefore should not be generalized beyond the tested parameters.

**Supplementary Information:**

The online version contains supplementary material available at 10.1038/s41598-026-36610-2.

## Introduction

The slab-column system, which consists of flat slabs, is widely used in the building of parking garages, high-rise buildings, ramps, warehouses, and hotels due to its many benefits, such as its ability to provide more interior space and to reduce time of construction^1–4^. However, the system is susceptible to early PS failure. The evidence of this lies in the significant damage and failures observed in SCJs during the earthquakes in Mexico and the Northridge in the 1980s and 1990s^5–9^. Moreover, in recent years, SCJs have frequently failed unexpectedly when subjected to vertical stresses as a result of construction negligence. The greatest safety risk associated with SCJs is thus early PS failure. Because standard concrete’s tensile strength is insufficient, PS failure frequently happens at SCJs. To prevent sudden failure, vertical transverse reinforcement or shear studs are occasionally installed at pivotal points of punching shears. However, excessive shear reinforcement will make construction more challenging, which could lower project quality and viability from an economic standpoint^10–12^. Increasing the thickness of the slab, using drop panels, or using column heads are other effective ways to make up for the low-tension concrete strength. But, these actions will result in a reduction in floor height while increasing structural self-weight and construction complexity^13^. Therefore, there is an urgent need for novel methods that can ensure the capacity and ductility of joints while maintaining ease of design and building.

Numerous investigations into the SCJs’ PS behavior have been done to date. Even though a lot of work has gone into understanding experimentally the mode failures, major influencing variables, and load-resisting techniques of SCJs^1,14^.In addition, several analytical equations have been created to calculate the PS capacity of SCJs^1,15,16^. In the meantime, a few studies have attempted to identify effective methods for preventing PS failure or enhancing the PS capacity of SCJs^1,17–20^. However, de-icing agent-induced corrosion of reinforcement in older parking garages and bridges has been largely ignored in previous research^1,2^.Reviewing the literature on the assessment of the mechanical behavior of SCJs in PS allows us to draw the conclusion that a higher corrosion ratio decreases PS capacity while increasing ultimate deflection^1,21^.

The corrosion techniques of RC elements have been thoroughly investigated^1^. As well as the impacts of corroded reinforcement on the characteristics of the concrete and steel, the mechanical performance of different corroded RC components has been estimated^22,23^. Because the natural corrosion procedure in RC buildings is normally relatively slow, the accelerated corrosion approach has been frequently utilized to expedite the corrosion procedure^24,25^. This method applies an electrochemical potential between an external cathode and a steel rebar that serves as the anode, with the corrosion ratio being managed by an impressed constant current density^1,26^.

Punching shear failure in slab–column joints is recognized as one of the most critical and brittle failure mechanisms in RC flat-slab systems. Unlike flexural failure, punching shear occurs suddenly with little warning and can trigger progressive collapse^27–30^. The susceptibility of slabs to PS failure is further amplified when reinforcement corrosion is present. Corrosion products cause internal expansion, leading to concrete cracking, reduction in bond strength, loss of dowel action, and deterioration of aggregate interlock three key mechanisms that normally contribute to punching shear resistance^2,31–34^. As a result, even moderate levels of corrosion can shift the failure mode from flexural to brittle punching shear and significantly reduce the residual capacity of existing structures. These concerns highlight the need for effective strengthening strategies, particularly for aging slabs exposed to aggressive environments.

Recent advancements in the field of RC structural rehabilitation have emphasized the importance of innovative composite materials and sustainable strengthening strategies to improve mechanical performance under various deterioration mechanisms^35–37^. Several studies have demonstrated that the integration of hybrid fiber-reinforced composites can significantly enhance confinement efficiency, stress–strain characteristics, and overall structural response in deteriorated concrete members^38–41^. Furthermore, improvements in the durability properties of cementitious materials achieved through recycled fibers, modified aggregates, or hybrid fiber systems highlight the increasing trend toward developing sustainable strengthening solutions^42–46^. These emerging materials offer enhanced tensile capacity, improved crack control, and superior resistance to environmental degradation, all of which are directly relevant to strengthening corroded slab–column joints where bond deterioration, reduced ductility, and premature brittle failures are key concerns^47–50^.

In parallel, advanced analytical and predictive approaches, including machine learning techniques, have been increasingly adopted to model the nonlinear behavior of strengthened concrete systems and to improve the accuracy of predicting residual strength under complex deterioration states^12,44,51^. Although the primary focus of the present study is the experimental and numerical assessment of punching shear performance in corroded RC slab–column joints strengthened with thin-ply hybrid FRP, the insights from these broader research efforts underline the growing importance of hybrid composites, sustainable materials, and advanced modeling tools in modern structural engineering practice^52–54^.

A review of recent literature on sustainable composite systems, recycled fiber materials, and hybrid confinement techniques such as those applied in eco-friendly mortars, FRP-confined concrete, and durable RC members further supports the relevance of hybrid glass/carbon FRP strengthening as an efficient solution for deteriorated slab–column systems^44^. These studies collectively reinforce the need to explore lightweight, high-performance, and durable strengthening approaches such as the one investigated in the present work.

Recent studies have also provided important insights into strengthening techniques for two-way slabs and slab–column systems under different loading and deterioration conditions. For example, several numerical and experimental investigations have explored innovative FRP systems, enhanced reinforcement layouts, and hybrid composite solutions to improve punching shear resistance and control brittle failures^55^. Additional research has examined the behavior of retrofitted two-way slabs strengthened with fiber composites and alternative reinforcement arrangements, demonstrating substantial improvements in stiffness and load capacity^56,57^. Other recent works have evaluated the effectiveness of strengthening techniques in slabs subjected to complex stress fields or deteriorated conditions, confirming the benefits of composite-based systems for enhancing performance^58^. These studies highlight the growing research focus on two-way slab strengthening and provide further motivation for investigating advanced FRP systems for corroded slab–column joints.

Despite significant research on strengthening RC slab–column joints, existing studies have mainly focused on traditional CFRP or GFRP systems and have not fully examined the potential of thin-ply hybrid composites or their performance under combined corrosion and punching shear action. Moreover, previous work often investigates either corrosion effects or strengthening techniques independently, without addressing their interaction on joint behavior. The present study advances the state of knowledge by introducing a novel strengthening technique based on thin-ply hybrid glass–carbon FRP strips arranged in a skewed configuration that more effectively intercepts radial punching cracks. Additionally, this work examines a realistic corrosion range (5–30%) and integrates experimental testing with validated FE modeling and a comprehensive parametric study. This combined framework provides deeper insight into how corrosion severity, FRP configuration, and strengthening parameters collectively influence punching shear behavior, clearly distinguishing this study from existing literature.

### Research importance and scope

Corrosion in the joint between column and slab in a RC flat slab system produces major problems, so some of these joints need to be strengthened. Also, the residual capacity of these corroded strengthened joints is especially important to the design engineer. After reviewing previous research, it was revealed that most existing studies have concentrated on RC beams, slabs, and columns and ignored the effect of corrosion on the joints or calculating the residual capacity of these joints. To fill this research gap, the PS behavior has been studied experimentally, and numerically.

## Experimental study

A detailed experimental program was conducted on eleven 1:3 scaled interior slab–column joint specimens. Each slab measured 900 mm × 900 mm with a thickness of 100 mm and a centrally cast column stub measuring 150 mm × 150 mm. The longitudinal reinforcement consisted of 8 mm bars arranged in orthogonal grids with a flexural reinforcement ratio of 0.92%. The corrosion level for the designated specimens was targeted at 15% using an accelerated impressed-current technique. Several strengthening schemes using GFRP, CFRP, and hybrid thin-ply FRP strips were applied externally at the tension surface.

All slabs were simply supported along their four edges using steel channels. Monotonic axial loading was applied through the column stub using a hydraulic actuator at a constant rate of 10 kN/min until failure. Central deflection was measured using an LVDT positioned beneath the column, while crack initiation and propagation were visually monitored throughout the test. The ultimate load, deflection at first crack, failure mode, and stiffness characteristics were recorded for all specimens.

### Details of specimens

**Fig. 1:**
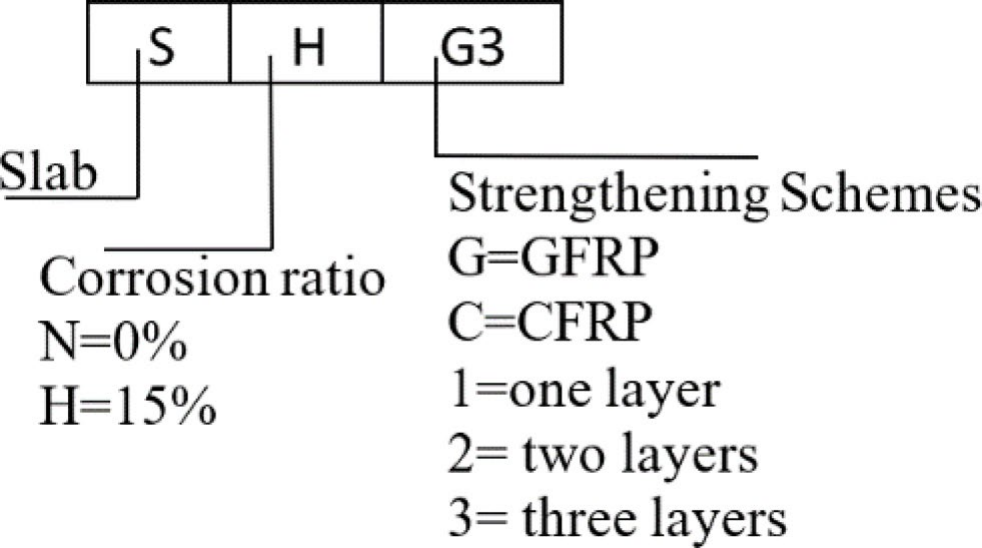
Specimen label.

**Fig. 2:**
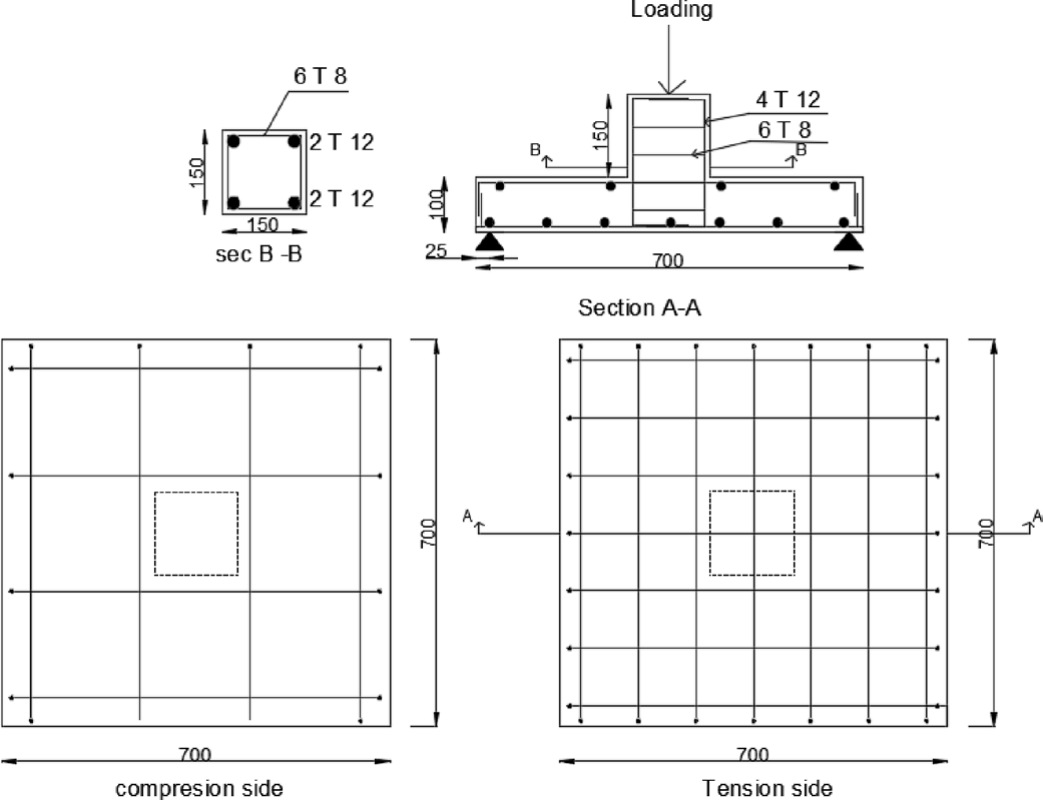
Schematic diagram of steel reinforcement arrangement.

This paper aimed to model an internal SCJ in a reinforced concrete structure, as seen in Fig. S1 (supplementary materials).The joint’s dimensions were selected based on bending moment contra flexure lines, located approximately at 0.2 times the center-to-center distance between columns according to CSA A23.3-19^59^.The specimens were labeled as illustrated in Fig. 1.Eleven scaled-down RC SCJ specimens (1:3 scale) with identical designs were constructed as listed in Table 1. All specimens had a flexural reinforcement ratio of 0.92%. Figure 2 depicts all dimensions and reinforcing configurations.

**Table 1:** Properties of specimens.

		Specimen code	Description of strengthening configuration	Nomenclature
Control		S-N	Control; no strengthening	S = Slab *N* = 0% corrosion ratio, H = 15% corrosion ratio, G = Glass Fiber Reinforced Polymer C = Carbon Fiber Reinforced Polymer num = number of layers 1 = one layer 2 = two layers 3 = three layers
		S-H	Corroded control; no strengthening	
External Strengthening	One type FRP	S-H-G1	Strengthened externally using 1 num GFRP	
		S-H-G2	Strengthened externally using 2 num GFRP	
		S-H-G3	Strengthened externally using 3 num GFRP	
		S-H-C1	Strengthened externally using 1 num CFRP	
		S-H-C2	Strengthened externally using 2 num CFRP	
		S-H-C3	Strengthened externally using 3 num CFRP	
	Hybrid	S-H-GCG	Strengthened externally using 3 num Hybrid	
		S-H-GC	Strengthened externally using 2 num Hybrid	
		S-H-GCC	Strengthened externally using 3 num Hybrid	

### Material properties

The measured 28th day average cubic compressive strength of concrete mixture was 30 N/mm^2^. The steel bars’ yield strength was 484 N/mm^2^, elongation was 15%, and ultimate strength was 624 N/mm^2^. Unidirectional FRP strips with dimensions of 50 mm width, 1.2 mm thickness, and a cross-sectional area of 60 mm^2^ were employed to strengthen the concrete slabs. The weight of the FRP strips was 1.6 g/cm^3^. The mechanical properties of the FRP strips and adhesive used in the strengthening application are provided in Table 2.

**Table 2:** Mechanical properties of the CFRP and GFRP sheets.

Type of FRP	Ultimate strength (*N*/mm^2^)	Ultimate strain (mm/mm)	Modulus of elasticity (GPa)
GFRP	511	0.0101	81.6
CFRP	621	0.012	231
Adhesive	24.8	0.010	4.5

### Accelerated corrosion method

**Fig. 3:**
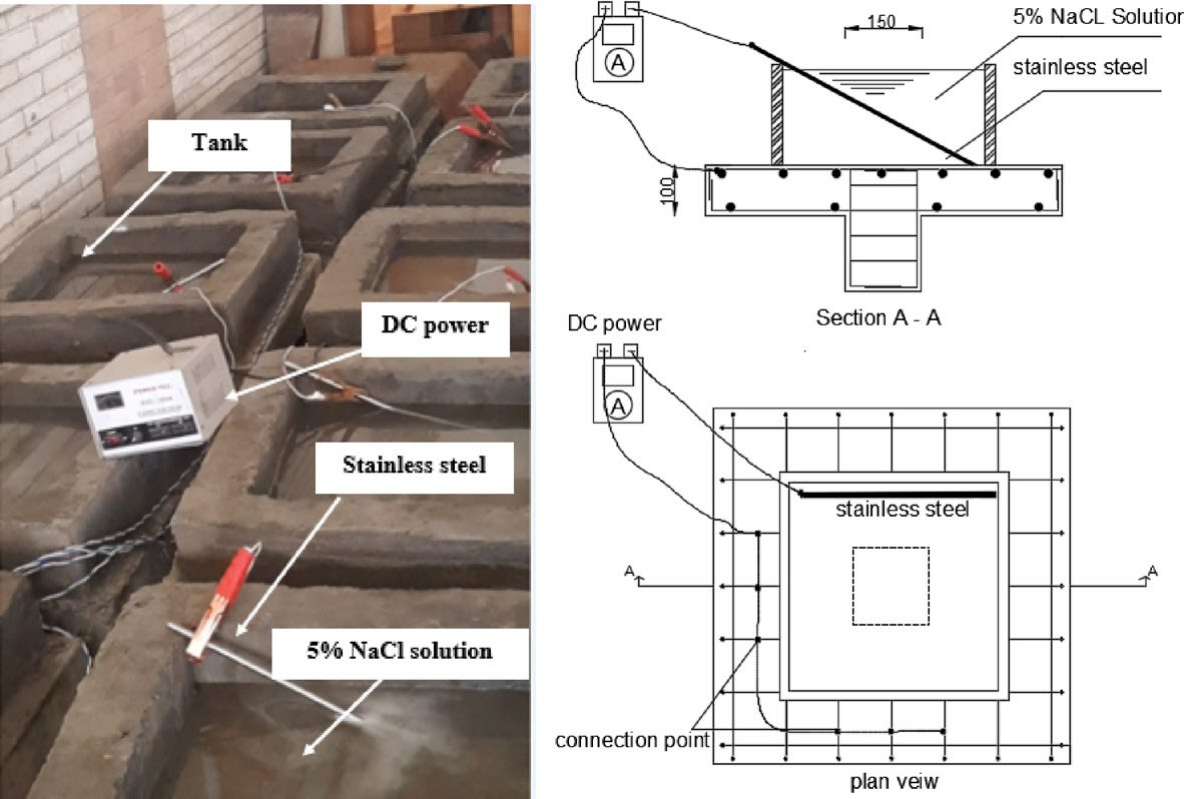
Method of accelerated corrosion.

In this study, an impressed-current accelerated corrosion technique was employed to induce controlled reinforcement corrosion in the RC slabs. A waterproof tank with internal dimensions of 450 mm × 450 mm was constructed around the column region of each specimen and filled with an electrolyte solution containing 3–5% sodium chloride (NaCl), which provides a chloride-rich environment capable of promoting corrosion. Prior to concrete casting, insulated wires were connected to the flexural reinforcement, which served as the anode during the corrosion process, while stainless-steel bars placed inside the electrolyte acted as the cathode and were connected to a regulated DC power supply, as shown in Fig. 3. Once the electrical current was applied, oxidation of the steel bars progressed at a controlled rate due to the chloride ions present in the electrolyte. The total duration required to reach the target corrosion level ranged from several days to several weeks depending on the desired mass loss. This duration was calculated using Faraday’s law^25,41,60^, which relates the applied current density, molar mass of iron, and ionic charge to the expected steel mass loss, as expressed in Eq. (1). After completing the corrosion period, loose rust products were removed and the actual corrosion ratio was verified through mass-loss measurements.

1 $$\:corrosion\:time\:\left(t\right)=\:\frac{Ionic\:charge\times\:Faraday\:constant\:\times\:masses\:of\:corrosion}{Iron\:molar\:mass\times\:current\:density\:of\:corrosion\:}$$

### Strengthening procedure

**Fig. 4:**
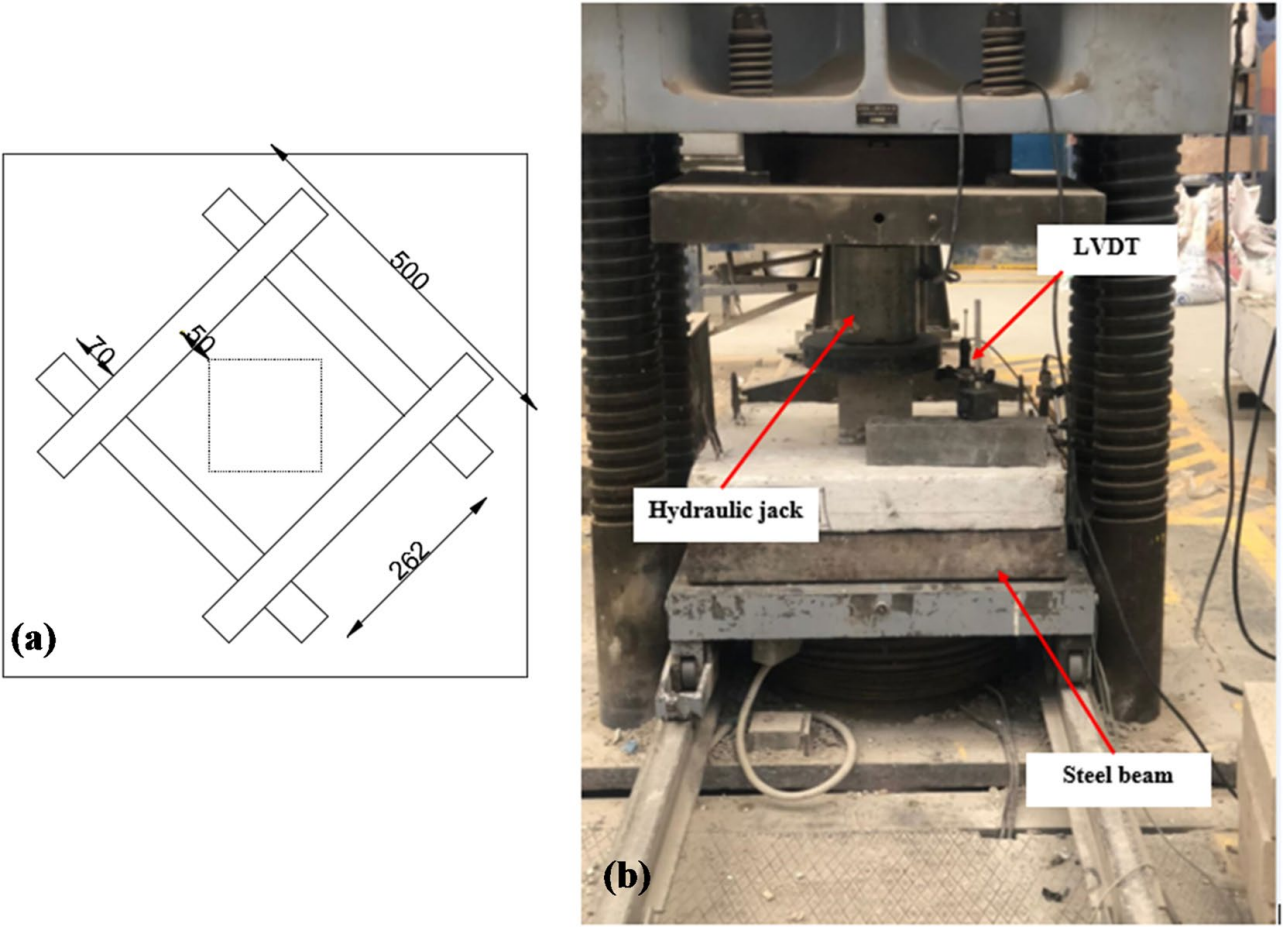
(**a**) Strengthening schemes, (**b**) test setup.

The unidirectional FRP strips were arranged in a skewed orientation for strengthened specimens, offset by 50 mm from the column face, as shown in Fig. 4a. Surface preparation involved removing the weak slab layer, cleaning with a high-pressure air jet, and applying epoxy adhesive. The strips were then pushed onto the concrete substrate, releasing trapped air with a roller. The FRP strips were placed on each other in the same direction.

### Test setup

The SCJs were supported by steel beams along all four edges to provide uniform simple support conditions, as illustrated in Fig. 4b. All specimens were tested under monotonic vertical loading applied concentrically through the column stub using a servo-controlled hydraulic actuator with a maximum capacity of 1000 kN. The load was applied at a constant rate of 10 kN/min to ensure stable and uniform loading throughout the experiment. A calibrated load cell integrated with the actuator continuously recorded the applied load, while a high-precision LVDT positioned directly beneath the column measured the central deflection of the slab during testing. Throughout the loading process, the development and propagation of cracks were carefully monitored. Punching shear failure was identified based on the formation of characteristic radial and circumferential cracks around the column perimeter, combined with the sudden loss of load-carrying capacity and the appearance of a conical failure surface. These visual and mechanical indicators were used to confirm the failure mode for each specimen.

## Experimental results and observations

### Corrosion status

**Fig. 5:**
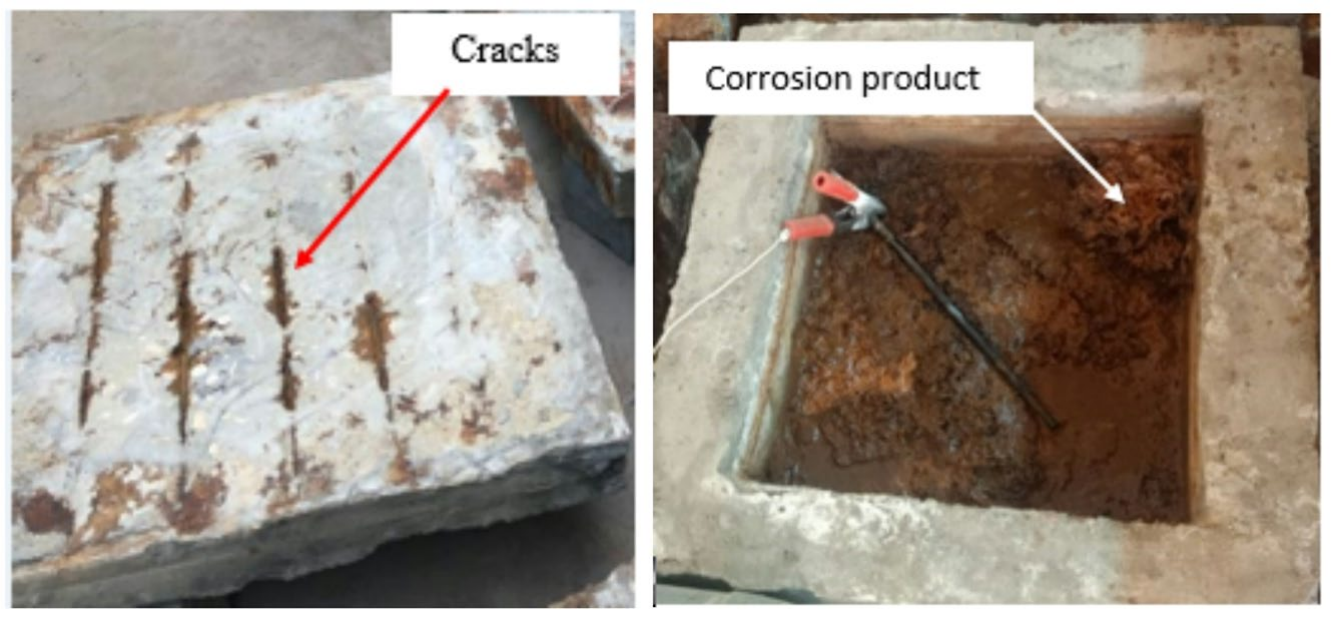
Corrosion cracks at the surface of the specimen S-H after the corrosion process.

As depicted in Fig. 5, a significant amount of corrosion product was observed on the specimen surface after accelerated corrosion was complete. Hairline cracks were detected in the slab after removing the corrosion products. Corroded rebars were removed from slabs and divided into seven coupons to calculate the average mass loss, as shown in Fig. S2 (supplementary materials). The steel coupons underwent cleaning, and the average mass loss of all corroded coupons was calculated to determine the rebar corrosion in each slab. The corrosion ratio was then computed using the following formula:

2 $$\:Corrosion\:ratio=\frac{mass\:of\:the\:original\:reinforcement-mass\:of\:the\:corroded\:reinforcement}{mass\:of\:the\:original\:reinforcement}\times\:100$$

The mass loss results are listed in Table 3. The visualized investigation of the surface of the corroded rebars in specimen S-H showed that there were localized pits of corrosion on the steel, although a uniform current was induced along the length of the corroded rebars^61,62^. Figure S3 (supplementary materials) indicates the corrosion ratio (CR) distributions for each reinforcement bar in each corroded specimen. The highest level of corrosion was found around the column perimeter. Due to the expansion force caused by corrosion, the concrete surrounding the column is more likely to crack than the concrete at the edge of the corroded zone. The properties of the corroded rebar were experimentally measured, as presented in Table 3.

**Table 3:** Actual corrosion ratio and material properties of corroded reinforcements.

Specimen code	Corrosion degree (%)		Average yield Strength (MPa)	Average ultimate Strength (MPa)	Average elongation (%)
	Average	Maximum			
S-H	15.3	18.3	392.2	505.6	12.7
S-H-G1	14.51	17	396.9	511.7	12.8
S-H-G2	15.55	17.9	390.7	503.7	12.7
S-H-G3	15.45	18.3	391.3	504.5	12.7
S-H-C1	14.75	17.3	395.5	509.9	12.8
S-H-C2	15.6	18	390.4	503.3	12.7
S-H-C3	15.35	18.1	391.9	505.2	12.7
S-H-GCG	14.95	16.1	394.3	508.3	12.8
S-H-GC	14.3	16.3	398.2	513.4	12.9
S-H-GCC	15.65	17.2	390.1	502.9	12.7

### Punching shear capacity

**Fig. 6:**
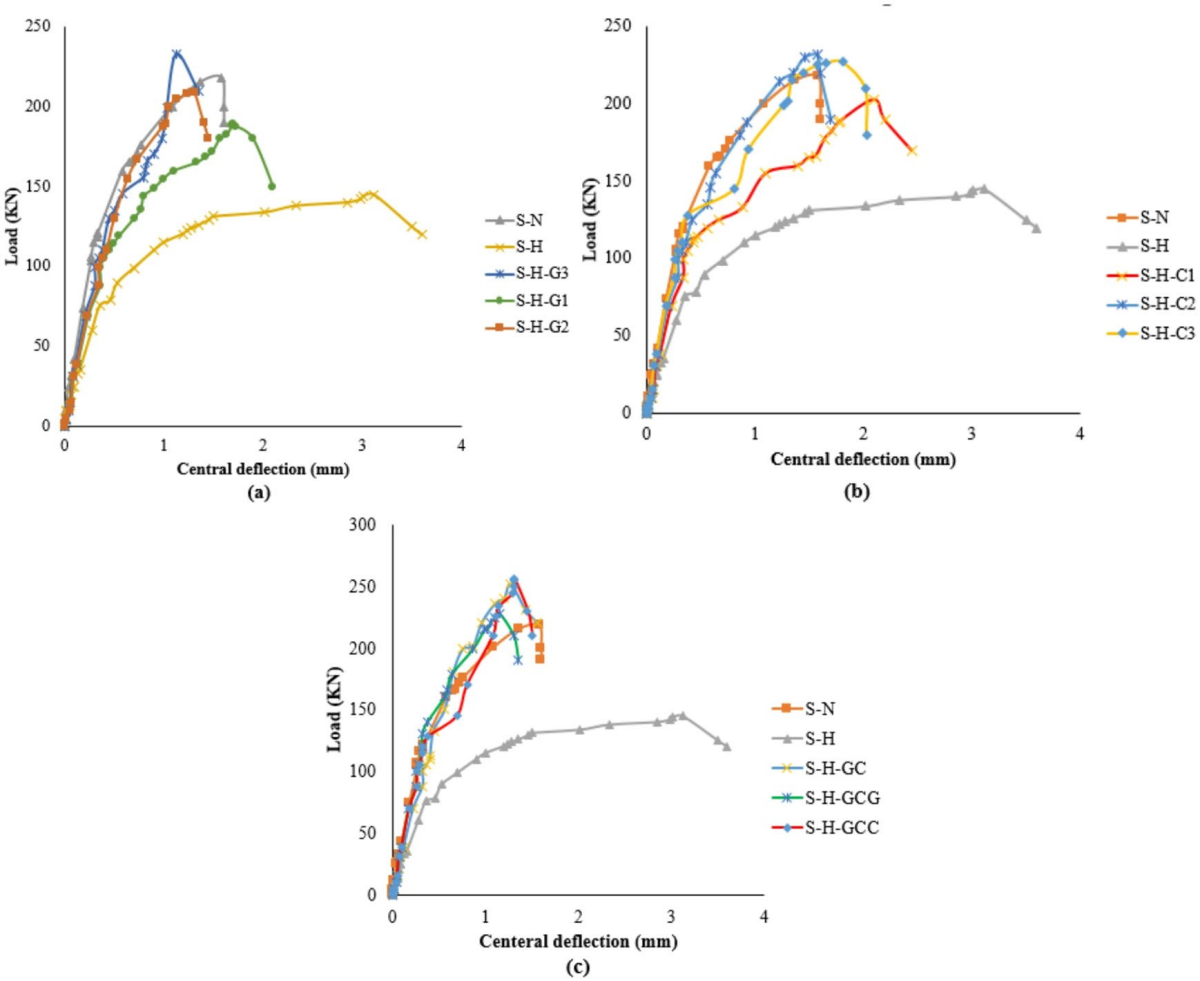
Load–deflection curves illustrating the punching shear behavior of slab–column joints: (a) specimens strengthened with GFRP systems; (b) specimens strengthened with CFRP systems; (c) specimens strengthened with hybrid glass/carbon FRP systems.

The load–deflection response of the specimens demonstrated clear behavioral stages influenced by corrosion and strengthening configuration. As shown in Fig. 6, the PS capacity of the SCJ decreased by 33.17%, while the ultimate deflection increased by approximately 99% when the corrosion ratio increased from 0% to 15%. In the corroded specimen, the initial linear-elastic stage was followed by an early deviation from linearity due to the premature formation of flexural cracks, reflecting the reduction in stiffness caused by weakened aggregate interlock, reduced dowel action, and loss of effective reinforcement area. These deterioration mechanisms significantly accelerated the stiffness degradation stage and led to a flexure–punching mixed failure at the final stage. In contrast, all strengthened specimens exhibited higher stiffness in the pre-cracking stage, a delayed onset of crack initiation, and enhanced load-carrying capacity compared to the corroded control (S-H). As summarized in Table 4, the PS capacity improvements achieved using GFRP, CFRP, and hybrid glass/carbon FRP systems were 29.66–51.03%, 40–60%, and 57.24–76.55%, respectively. Strengthened specimens also showed a more gradual transition into the stiffness degradation phase and a more stable load–deflection behavior, although failure ultimately occurred through punching shear or punching accompanied by partial FRP debonding. These observations confirm that the FRP systems, particularly hybrid arrangements, effectively mitigated the adverse effects of corrosion and improved overall structural performance.

**Table 4:** Summary of test results.

Specimen	Vcr (kN)	Dcr (mm)	Vu (kN)	Du (mm)	% Change in Vu vs. S-H	Ki (kN/mm)
S-N	44.62	0.71	217	1.53	+ 49.66%	62.8
S-H (Corroded Control)	40.64	1.01	145	3.1	0.00%	40.2
S-H-G1	70	0.98	188	1.73	+ 29.66%	71.5
S-H-G2	72	0.98	209	1.31	+ 44.14%	73.5
S-H-G3	70	0.98	219	1.13	+ 51.03%	71.5
S-H-C1	89	1.02	203	2.10	+ 40.00%	87.3
S-H-C2	121	1.01	232	1.58	+ 60.00%	119.9
S-H-C3	120	1.02	227	1.81	+ 56.55%	117.7
S-H-GCG	121	1	228	1.15	+ 57.24%	121.0
S-H-GC	121	1.02	252	1.28	+ 73.79%	118.7
S-H-GCC	122	1.03	256	1.31	+ 76.55%	118.5

Specimen	Ku (kN/mm)	Ductility index	Pre-cracking energy (kN·mm)	Post-Cracking Energy (kN·mm)	Total Energy (kN·mm)	Failure mode
S-N	210.22	1.00	15.84	157.76	173.60	Flexure
S-H (Corroded Control)	49.93	2.03	20.52	246.28	266.80	Flexure/ Punching
S-H-G1	157.84	1.13	34.30	188.56	222.86	Punching
S-H-G2	419.27	0.85	35.28	148.32	183.60	Punching/ Debonding
S-H-G3	996.20	0.74	34.30	128.92	163.22	Punching/ Debonding
S-H-C1	105.56	1.37	45.39	261.21	306.60	Punching
S-H-C2	194.74	1.03	61.01	217.86	278.87	Punching
S-H-C3	135.44	1.18	61.20	252.84	314.04	Punching/ Debonding
S-H-GCG	705.26	0.75	60.50	140.47	200.97	Punching/ Debonding
S-H-GC	495.07	0.84	61.71	177.87	239.58	Punching
S-H-GCC	484.18	0.85	62.83	184.15	246.98	Punching

V_cr_ is the load at first crack; D_cr_ is the deflection atfirst crack load; V_u_ is the ultimate load; and D_u_ is the deflection at the ultimate load.

### Effect of strengthening parameters on structural performance indicators

To enable a comprehensive assessment of the mechanical response of all specimens, the consolidated performance table (Table 4) summarizes both the experimental measurements and the derived structural indicators. These include the ultimate load and its corresponding deflection, the percentage increase in punching shear capacity relative to the corroded control specimen (S-H), the initial stiffness (Ki) defined from the linear-elastic segment of the load–deflection curve, the post-cracking stiffness (Ku) representing the slope after first crack formation, and the pre- and post-cracking energy absorption obtained from the area under the load–deflection relationship.

Together, these metrics provide a clear picture of how the key strengthening parameters—FRP type, number of layers, thickness, and strip orientation affect both strength and deformation behavior. The results demonstrate that hybrid glass–carbon FRP systems consistently deliver the highest gains in load capacity, initial stiffness, and overall energy absorption, confirming their superior ability to intercept radial punching cracks and delay stiffness degradation. Moreover, the reduction in post-cracking deformations observed in the strengthened specimens highlights the beneficial role of FRP systems in improving toughness and mitigating the adverse effects of corrosion. Overall, the combined indicators reveal that hybrid configurations provide the most balanced enhancement in strength, stiffness, and ductility among all tested strengthening schemes.

### Failure mode

**Fig. 7:**
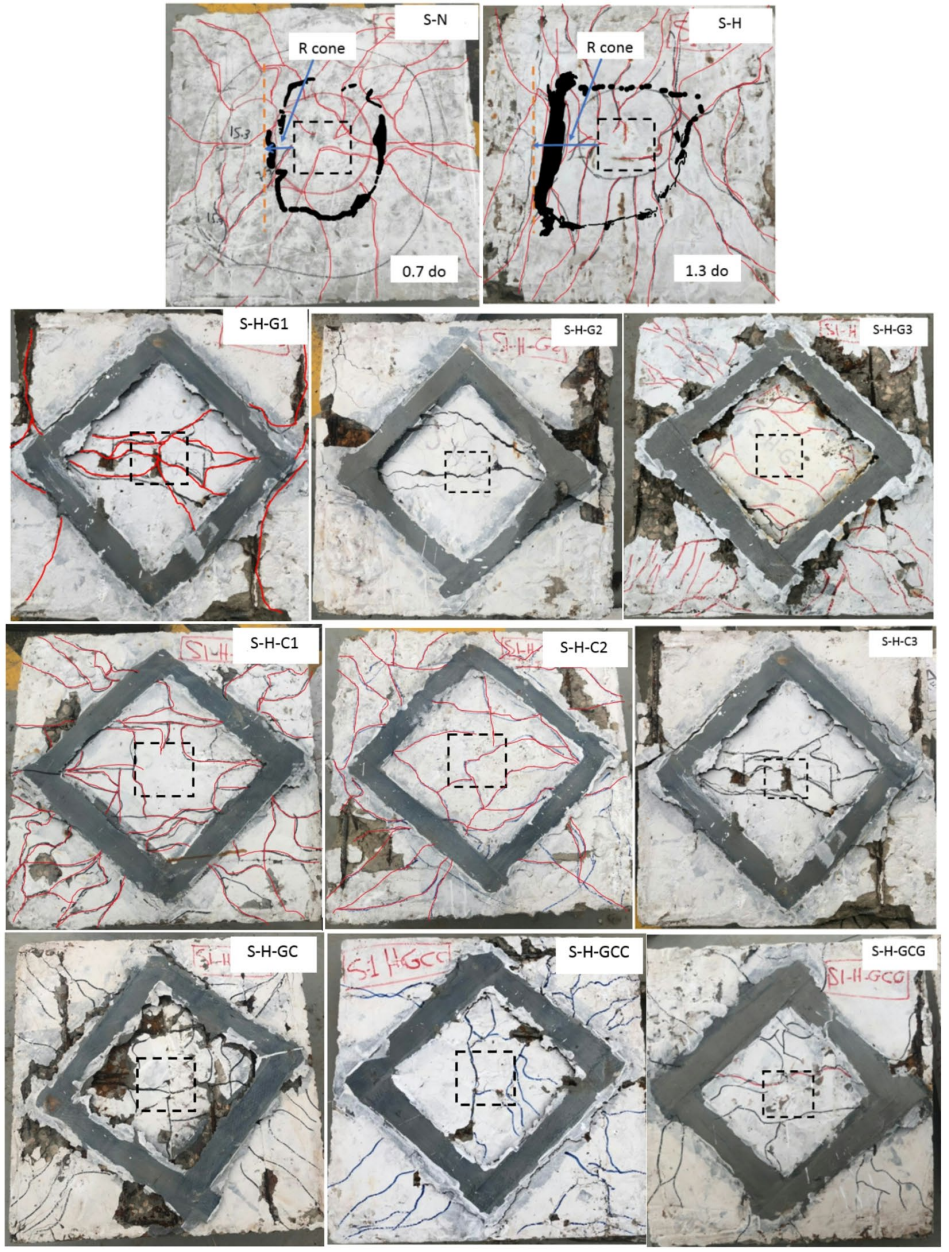
Crack patterns and critical failure zones of strengthened specimens.

The control specimen (S-N) failed in flexure, while the corroded reference specimen (S-H) failed in flexure/punching failure. In all strengthened specimens, the common mode of failure was punching shear, as depicted in Fig. 7. However, the specific failure patterns varied slightly among the different slabs. In the strengthened specimens (S-H-G1, S-H-C1, S-H-C2, S-H-GC, and S-H-GCC), flexural cracks initiated near the mid span and then spread towards the edges of the slab. As loading increased, large splitting cracks in the tension side were observed, and sudden concrete spalling followed. Flexural cracks initiated may be due to the bottom steel reinforcing bars being completely exposed due to a lack of stress sharing with the concrete and FRP. After further concrete splitting was observed, typical PS failure commenced. The concrete located near the punching cone was split along the reinforcing bars due to the concentrated pressure applied by the column stub, as shown in Fig. 7. Failure of other strengthened specimens(S-H-G2, S-H-G3, S-H-C3, and S-H-GCG) was identified as PS failure with debonding of FRP. The FRP sheets contributed to the increase in the load capacity until the bond between the FRP sheet and the concrete failed. Localized debonding cracks appeared at a later stage of loading due to the development of the intermediate flexural cracks and the diagonal shear cracks, which resulted in a separation of the strengthening materials. These cracks were located along the edges of the FRP strip length. After the formation of these cracks, the specimen failed due to the rapid development of the critical shear crack after the FRP debonded from the slab without any observed rupture of the FRP material, which shows that the FRP sheets did not reach their maximum tensile strength, as shown in Fig. 7.

### Stiffness, ductility, and energy dissipation

The results show that for unstrengthening specimens, the increase in the SCJ’s corrosion ratio resulted in a reduction in its initial stiffness (Ki) and a significantly lower ultimate stiffness (Ku). It is noted that ki considerably increased for GFRP or CFRP or hybrid-strengthened SCJs compared to corroded control by 77.8–82.8%, 111.6–198.2%, and 194.7–200.9%, respectively, as shown in Table 4. The experimental findings indicate that as the CR increases, the ultimate deflection also increases, leading to a rise in the ductility index with higher corrosion ratios.

The energy-dissipating capacity increases with the increase of CR because of more ductile failure, as shown in Table 4. It can be observed that the energy dissipation values for the strengthened specimens are generally lower compared to the control and corroded reference specimens. This is because the addition of FRP composites can increase the stiffness of the structure, which can reduce its ability to undergo deformation and dissipate energy. Among the strengthened specimens, it can be observed that the ductility index and energy dissipation values generally decrease with increases in the number of FRP layers. Additionally, the use of hybrid FRP composites can provide higher stiffness, lower ductility index values, and energy dissipation compared to the use of single FRP composites.

## Numerical study

Numerical simulations of corroded SCJs’ performance under various strengthening techniques have been developed using ABAQUS software.

### Finite element modeling

#### Elements and materials

In this study, numerical FE models were developed using various element types to represent different components of the structure. Stirrups were modeled using two-node elements (T3D2), while concrete and longitudinal reinforcement bars were represented using three-dimensional solid elements (C3D8R). FRP sheets were simulated using triangular elements of the 6-node in plane shell (SC6R), and adhesives were modeled using three-dimensional cohesive elements of the 6-node (COH3D6)^63^.

In the finite element analysis, material properties and constitutive models were defined carefully to replicate the experimental behavior. Concrete was modeled using the Concrete Damage Plasticity (CDP) model, where compressive and tensile stress–strain relationships, dilation angle, viscosity parameter, and damage coefficients were assigned according to validated values from the literature and the experimental results. Steel reinforcement was modeled as an elastic–plastic material with experimentally measured yield strength, ultimate strength, elastic modulus, and corrosion-adjusted reductions applied where relevant. FRP layers were defined as linear elastic orthotropic materials, while adhesive and FRP concrete interfaces were represented using cohesive zone elements with traction separation laws calibrated from available test data.

Boundary conditions were applied to match the experimental test configuration. All slab edges were restrained vertically to simulate simple supports, while in-plane movement remained free to avoid artificial stiffness. Vertical load was applied at a reference point coupled to the top of the column stub to ensure uniform load transfer, consistent with the hydraulic actuator used experimentally.

A mesh sensitivity analysis was performed to determine the optimal element size. Several mesh densities were evaluated, ranging from coarse (40 mm) to fine (15 mm), particularly around the punching region. Results showed that refining the mesh improved crack localization and load–deflection accuracy. An element size of approximately 20 mm in the critical punching zone, transitioning to a coarser mesh toward the slab edges, provided stable convergence with less than 3% variation in ultimate load predictions relative to the finest mesh. This configuration was adopted in all models.

The Concrete Damage Plasticity (CDP) Model was employed to define material properties. This model requires five plastic damage factors listed in Table S1 (supplementary materials)^64^. The computed damage-plasticity parameters are presented in Fig. S4 (supplementary materials)^65^.The detailed finite element modeling for the conducted numerical analysis can be found in Ahmed et al. ^2^ and (supplementary materials).

#### Reduction in the properties of the concrete and rebar

The study considers the effects of corrosion on reinforcing steel, taking into account the reduction in the properties of both steel and concrete. Equations (3) to (6), sourced from Kai Qian^21^ and Ahmed^2^, are used to estimate the reduction in mechanical properties of the corroded joints, up to a 30% CR, in a numerical parametric study. The accuracy of the calculated values from these equations is verified by comparing them with laboratory results, showing good agreement, as demonstrated in Table S2 (supplementary materials).

3 $$\:{f}_{cu,c}={(1\:+\:K\frac{{{\upepsilon\:}}_{t}}{{{\upepsilon\:}}_{0}}\:\:\:)}^{-1}{\times\:f}_{cu}$$

4 $$\:{f}_{yc}=(1\:-\:\alpha\:1(CR/100)\times\:{f}_{y}$$

5 $$\:{f}_{uc}=(1\:-\:{\alpha\:}_{1}(CR/100)\times\:{f}_{u}$$

6 $$\:{E}_{sc}=(1\:-\:{\alpha\:}_{2}(CR/100)\times\:{E}_{S}$$

In the equation provided,$\:{f}_{cu}$represents the concrete compressive strength, $\:K$represents a coefficient that bases on the roughness and the diameter of the rebar ($\:K$is assigned a value of 0.1for ribbed bars of moderate diameter^63^, and$\:{\:{\upepsilon\:}}_{0}$represents the strain value at the ultimate $\:{f}_{cu}$; $\:{{\upepsilon\:}}_{t}$ is the average tensile strain in the cracked concrete normal to the direction of the applied compression^63^. Furthermore,$\:{\alpha\:}_{1}$ and$\:{\alpha\:}_{2}$refer to empirical factors with values of 1.24 and 0.75, respectively, in the case of uniform corrosion of rebars, and $\:CR$stands for the corrosion ratio, while$\:{E}_{s}$ and $\:{f}_{y}$refer tothe elastic modulus and yield strength of the uncorroded rebars, respectively.

#### Reduction in bond strength

In the context of corroded reinforcements in structural concrete joints (SCJs), the assumption of perfect bond, which applies to uncorroded SCJs, becomes invalid. Previous studies have explored various techniques to model the concrete-steel bond for FE analysis^1,2,66^. ABAQUS provides several options for simulating the bond for both corroded and uncorroded SCJs, with one of the commonly used options being surface interaction techniques. In this study, a surface-based cohesive behavior was employed to simulate the bond in RC SCJ. The complete bond behavior, which can be employed to approximate the bond-slip curve between traction (bond stress) and separation (slip), is illustrated in Fig. S5 (supplementary materials)^2,63,66,67^.The surface-based cohesive method introduced in this study relies on specific parameters such as maximum bond strength ($\:{\tau\:}_{max}$), maximum separation ($\:{S}_{max}$), and bond stiffness ($\:{K}_{nn},\:{K}_{tt},\:and\:kss$). In ABAQUS, the reduced bond strength ($\:{{\uptau\:}}_{\mathrm{m}\mathrm{a}\mathrm{x}}^{R}$) due to rebar corrosion is determined using Eq. (7)^63^. To consider the effect of rebar corrosion, a reduction factor ($\:R$) is employed, following Eq. (8) as proposed by Maaddawy^25^.

7 $$\:{{\uptau\:}}_{\mathrm{m}\mathrm{a}\mathrm{x}}^{R}=\:R(0.55\:+\:0.24\frac{{c}_{c}}{{d}_{b}})\surd\:fc^{\prime\:}+\:0.191\frac{{A}_{t}{F}_{yt}}{{S}_{s}{d}_{b}}$$

8 $$\:R\:=\:(A1+\:A2(\frac{CR}{100}\left)\right)$$

In the equations provided, $\:{A}_{t}$represents thetotal area of the transverse reinforcement, c_c_denotes the smaller value between the clear concrete cover and half of the clear spacing between rebars, $\:{d}_{b}$ is the diameter of the rebar, $\:{S}_{s}$stands for the spacing of the transverse reinforcement, $\:{F}_{yt}$represents the yield stress of the transverse reinforcement. The maximum slip ($\:{S}_{max}$) and bond stiffness values should be estimated by Eqs. (9), (10), and (11)^63^. Equations (7)–(11) were validated with experimental results in Table S3 (supplementary materials) and used for the numerical analyze.

9 $$\:{S}_{max}=\:0.15{C}_{o}{e}^{\frac{10}{3}ln\left(\frac{{{\uptau\:}}_{\mathrm{m}\mathrm{a}\mathrm{x}}^{R}}{\tau\:1}\right)}+\:{S}_{o}ln\:\left(\frac{{\tau\:}_{1}}{{{\uptau\:}}_{\mathrm{m}\mathrm{a}\mathrm{x}}^{R}}\right)$$

10 $$\:{K}_{ss}=\:{K}_{tt}=\frac{{{\uptau\:}}_{\mathrm{m}\mathrm{a}\mathrm{x}}^{R}}{{S}_{max}}$$

11 $$\:{K}_{nn}=\:100{K}_{tt}=\:100kss$$

where $\:{C}_{o}$ is the rib spacing, $\:{\tau\:}_{1}=2.57\sqrt{f\mathrm{c}^{\prime\:}}$, ′=0.8 $\:{f}_{cu}$, $\:{S}_{o}$ equal to 0.4 mm for confined concrete.

#### FRP/concrete interface

The FRP was considered a linear elastic material, and its parameters were outlined in Table 2. In this study, a cohesive model was employed to characterize the bond condition for the interface between FRP and adhesive, as well as the contact between adhesive and concrete. Input parameters for the cohesive model, such as initial stiffness related to adhesive properties, shear strength, and fracture energy, were modeled as functions of the tensile strength of concrete and adhesive properties. The adhesive/concrete interface’s mechanical behavior was represented as a correlation between local stress and relative displacement, utilizing the simplified bilinear bond-slip model^68^, illustrated in Fig. S6 (supplementary materials).

A damage evolution law was established, allowing the bond-slip curve to unload linearly from the origin once the interface enters the softening range. The maximum bond/shear stress experienced by the interface ($\:{\tau\:}_{max}$) and the corresponding slip when the bond stress reaches the maximum ($\:{S}_{o}$) are determined by a width ratio parameter ($\:{\:\beta}_{w\:}$) and the tensile strength of the concrete. The linear part of the curve is represented by the $\:{\mathrm{K}}_{nn}$, $\:{\mathrm{K}}_{ss}$, and $\:{\mathrm{K}}_{tt}$parameters in the Interaction/ contact property/cohesive behavior ofthe software. The $\:{\mathrm{K}}_{nn}$ value is equal to the elasticity modulus of theconcrete. The expressions for the maximum local bond strength ($\:{\tau}_{max}$), corresponding slip ($\:{S}_{o}$), initial stiffness and total fracture energy are as follows (Eqs. 12–16).

12 $$\:{\tau\:}_{max}=1.5{\:\beta\:}_{w\:}{f}_{t}\:$$

13 $$\:{S}_{o}=0.0195\:{\beta\:}_{w}{f}_{t}$$

14 $$\:{\:\beta\:}_{w\:}=\sqrt{\frac{2.25-{b}_{f}/{b}_{c}}{1.25+{b}_{f}/{b}_{c}}}\:$$

15 $$\:{K}_{ss}{=\:K}_{tt}=\frac{{\tau\:}_{max}}{{S}_{o}}$$

16 $$\:{G}_{f}=0.308{\:\beta\:}_{w}^{2}{\sqrt{f}}_{t}$$

Where $\:{b}_{f}$ is the width of the FRP (mm), and$\:{b}_{c}$ is the width of a concrete substrate (mm).

The interfacial fracture energy is minimally influenced by the FRP stiffness but is significantly affected by the mechanical properties of the concrete and to a lesser extent, the adhesive properties^69^. Debonding of the FRP strips is represented by the onset of damage in the cohesive elements^70^. Damage initiation is defined using a maximum nominal stress criterion as described by ^71,72^.

### Finite element model validation

**Fig. 8:**
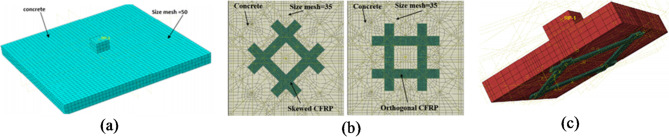
Finite element models, (**a**) Quin et al.^1^, (**b**) M.A.L. Silva et al.^73^, and (**c**) experimental work.

**Fig. 9:**
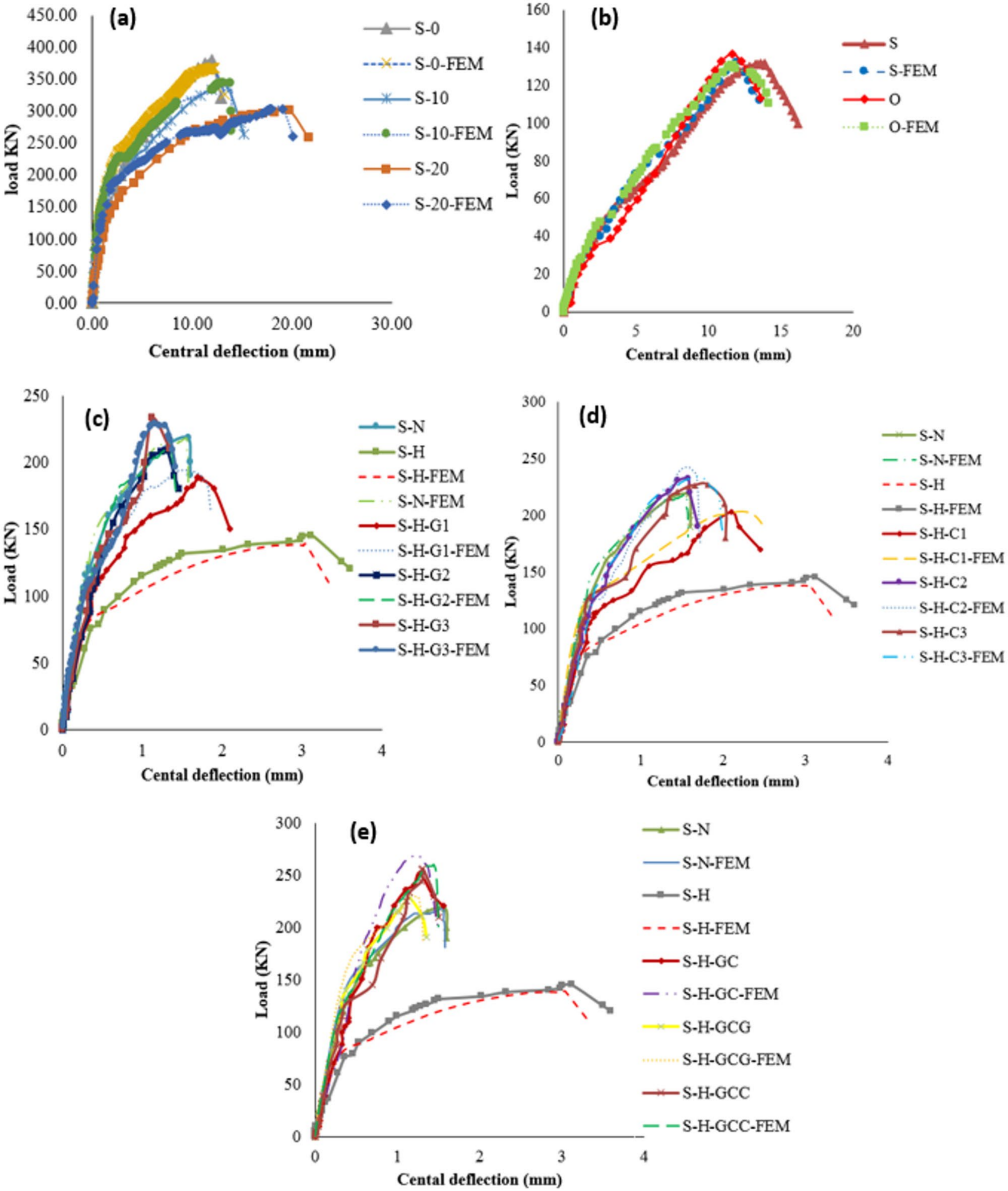
Load-deflection curves of experimental and FE simulation results, (**a**) Quin et al.^1^, (**b**) M.A.L. Silva et al.^73^, (**c**) GFRP strengthening, (**d**) CFRP strengthening, and (**e**) Hybrid strengthening.

**Fig. 10:**
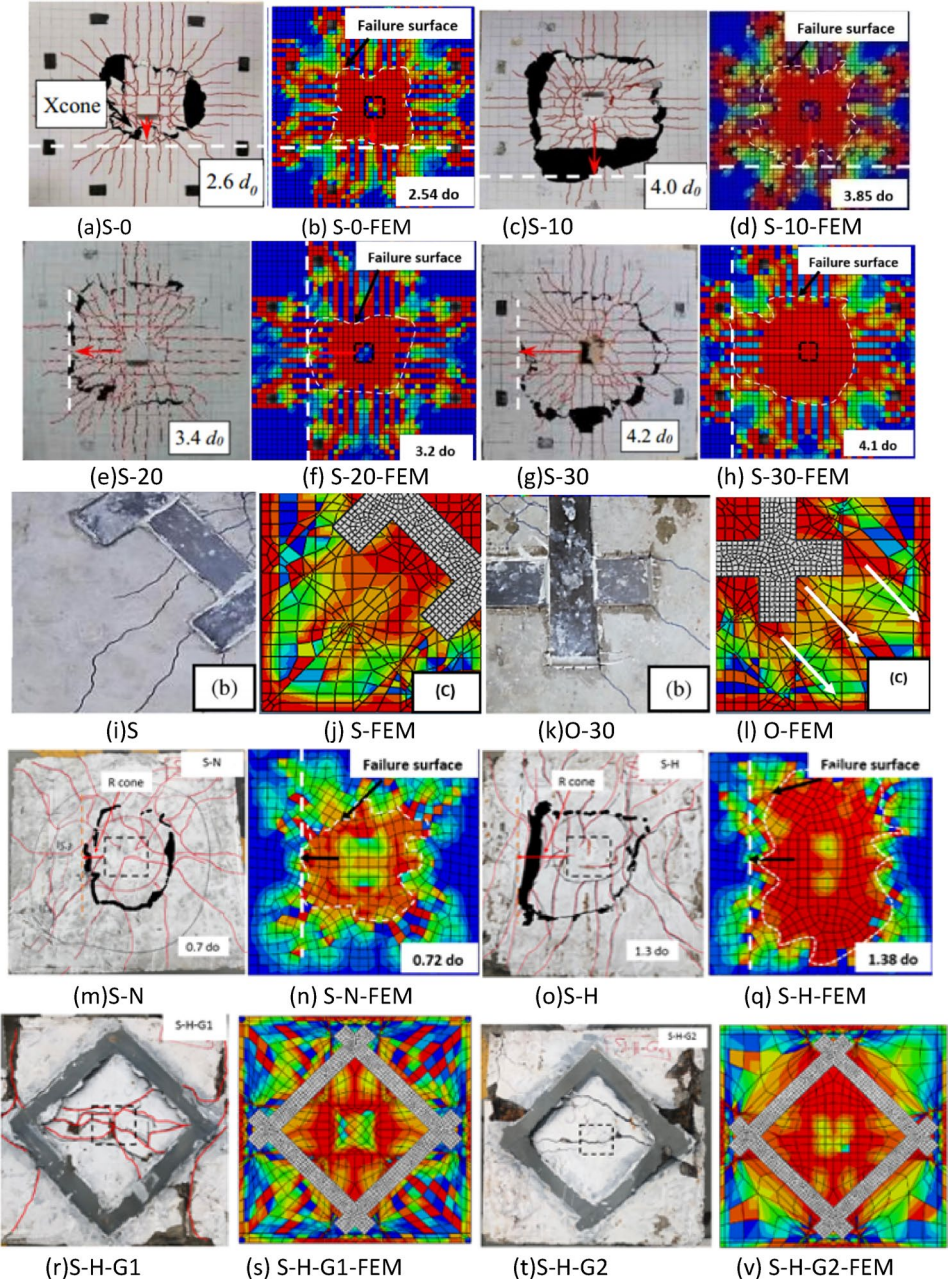

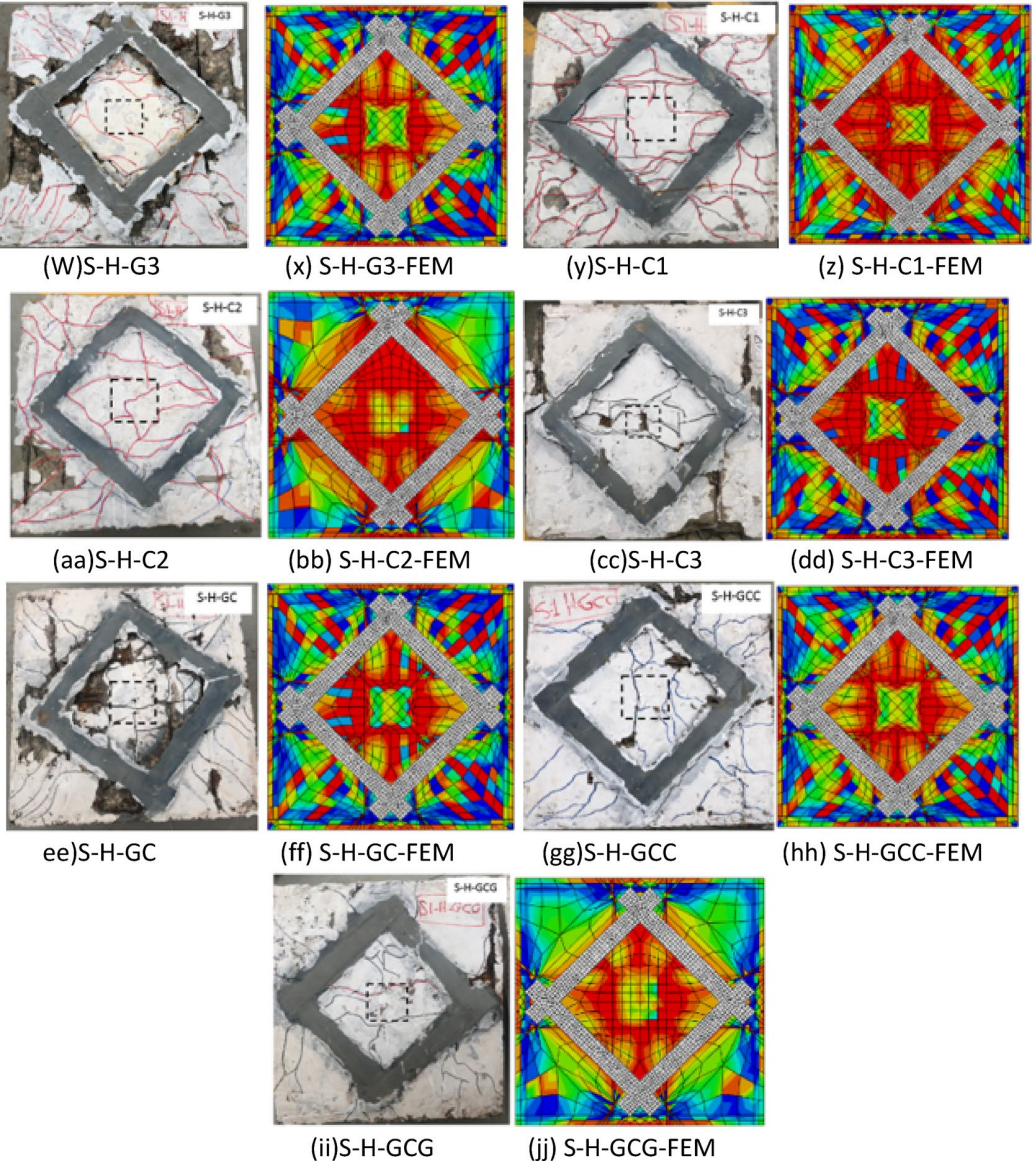
Comparison of test and simulation failure modes, (**a**) S-0 (Quin et al.^1^), (**b**) S-0-FEM (Quin et al.^1^), (**c**) S-10 (Quin et al.^1^), (**d**) S-10-FEM(Quin et al.^1^), (**e**) S-20 (Quin et al.^1^), (**f**) S-20-FEM (Quin et al.^1^), (**g**) S-30 (Quin et al.^1^), (**h**) S-30-FEM (Quin et al.^1^), (**i**) S (M.A.L. Silva et al.^73^), (**j**) S-FEM (M.A.L. Silva et al.^73^), (**k**) O-30 (M.A.L. Silva et al.^73^), (**l**) O-FEM (M.A.L. Silva et al.^73^), (**m**) S-N (experimental work), (**n**) S-N-FEM (experimental work), (**o**) S-H (experimental work), (**q**) S-H-FEM (experimental work), (**r**) S-H-G1 (experimental work), (**s**) S-H-G1-FEM (experimental work), (**t**) S-H-G2 (experimental work), (**v**) S-H-G2-FEM (experimental work), (**W**) S-H-G3 (experimental work), (**x**) S-H-G3-FEM (experimental work), (**y**) S-H-C1 (experimental work), (**z**) S-H-C1-FEM (experimental work), (**aa**) S-H-C2 (experimental work), (**bb**) S-H-C2-FEM (experimental work), (**cc**) S-H-C3 (experimental work), (**dd**) S-H-C3-FEM (experimental work), (**ee**) S-H-GC (experimental work), (**ff**) S-H-GC-FEM (experimental work), (**gg**) S-H-GCC (experimental work), (**hh**) S-H-GCC-FEM (experimental work), (**ii**) S-H-GCG(experimental work), and (**jj**) S-H-GCG-FEM (experimental work). (**a**) S-0, (**b**) S-0-FEM, (**c**) S-10, (**d**) S-10-FEM, (**e**) S-20, (**f**) S-20-FEM, (**g**) S-30, (**h**) S-30-FEM, (**i**) S, (**j**) S-FEM, (**k**) O-30, (**l**) O-FEM, (**m**) S-N, (**n**) S-N-FEM, (**o**) S-H, (**q**) S-H-FEM, (**r**) S-H-G1, (**s**) S-H-G1-FEM, (**t**) S-H-G2 (**v**) S-H-G2-FEM, (**W**) S-H-G3, (**x**) S-H-G3-FEM, (**y**) S-H-C1 (**z**) S-H-C1-FEM (**aa**) S-H-C2, (**bb**) S-H-C2-FEM, (**cc**) S-H-C3 (**dd**) S-H-C3-FEM (**ee**) S-H-GC, (**ff**) S-H-GC-FEM, (**gg**) S-H-GCC (**hh**) S-H-GCC-FEM, (**ii**) S-H-GCG (**jj**) S-H-GCG-FEM.

Finite element analysis has been conducted using Abaqus and validated by three experimental programs: Quin, et al.^1^ to validate modeling rebars corrosionon a large scale, M.A.L. Silva, et al.^73^ to validate modeling of strengthening technique, and eleven experimental specimens in the current experimental study to validate both corrosion and strengthening technique in a small scale. Figure 8 shows the FE model of all validation models constructed. The simulated and load-deflection curves of the specimens are compared in Fig. 9. As evidenced by Table 5, the FE model employed in this study accurately predicts the PS capacity and ultimate displacement of the RC joints. Figure 10 presents a comparison between the modeled failure modes and the actual failure modes. The results of the FE model closely align with the measurements, as demonstrated in Fig. 10. Therefore, this study’s FE model is appropriate for predicting the PS failure behavior of corroded strengthened joints and is used in the subsequent extended analysis.

**Table 5:** Comparison of ultimate load (kN) and ultimate Deflection (mm) between experimental and FE modeling.

Reference	Specimen code	Ultimate load, Vu (kN)			Central deflection at Vu (mm)		
		Exp	FE	Accuracy	Exp	FE	accuracy
Quin, et al.^1^	S-0	379	367.61	3.12	11.92	12.23	− 2.67
	S-10	337	344.74	− 2.14	13.58	13.78	− 1.51
	S-20	301	304.24	− 0.76	19.74	19.01	3.69
	S-30	289.	303.88	− 4.84	22.13	21.86	1.18
M.A.L. Silva et al.^73^	S	132.16	129.57	1.96	11.93	12.85	− 7.78
	O	130.31	136.62	− 4.84	11.69	11.04	5.58
Experimental specimens	S-N	217	217.5	− 0.24	1.55	1.56	− 0.41
	S-H	145	138.6	4.40	3.12	3.04	2.57
	S-H-G1	188	194.0	− 3.17	1.73	1.62	6.23
	S-H-G2	209	209.7	− 0.33	1.31	1.23	5.87
	S-H-G3	219	228.0	− 4.11	1.13	1.16	− 2.69
	S-H-C1	203	202.8	0.08	2.10	2.23	− 6.19
	S-H-C2	232	242.0	− 4.31	1.58	1.58	0.00
	S-H-C3	227	233.0	− 2.64	1.81	1.72	4.97
	S-H-GC	228	269.0	− 17.98	1.15	1.24	− 7.67
	S-H-GCG	252	231.0	8.33	1.28	1.19	7.36
	S-H-GCC	256	260.0	− 1.56	1.31	1.44	− 10.20

### Parametric investigation

In this study, validated FE models have been conducted to investigate the effect of different parameters on the behavior of corroded joints. Ahmed et al.^2^ previously examined several parameters on this scale model, including corrosion ratio, corrosion area, column rectangularity, slab thicknesses, reinforcement ratio, concrete compressive strength, steel yield strength, and supporting distance. Full-scale models of the specimens at this scale were also examined^2^, and the results indicated that there was no size effect for these parameters in the full-scale model. The effective parameters considered in this study are strengthening configurations with different tensile reinforcement ratios, number of layers, FRP thickness, location of FRP from the column face, and strengthening schemes, as shown in Table 6. In the parametric study, the reference corroded specimen is represented by the S-H specimen, while the reference strengthened specimen is represented by the S-H-C1 specimen.

**Table 6:** Models description.

Factors	Specimen ID	Number of layers	FRP thickness (mm)	FRP location from column face (mm)	Strengthening configurations
Low tensile reinforcement ratio (0.5%)	S-H-0.5 (corroded control)	–	–	–	–
	S-0.5	1	1.2	50	Skewed FRP sheets
	O-0.5	1	1.2	50	orthogonal FRP sheets
	O-R-0.5	1	1.2	0	orthogonal-radial FRP sheets
	S-R-0.5	1	1.2	0	skewed-radial FRP sheets
Medium tensile reinforcement ratio (0.92%)	S-H-0.92 (corroded control)	–	–	–	–
	S-0.92	1	1.2	50	skewed FRP sheets
	O-0.92	1	1.2	50	orthogonal FRP sheets
	O-R-0.92	1	1.2	0	orthogonal-radial FRP sheets
	S-R-0.92	1	1.2	0	skewed-radial FRP sheets
High tensile reinforcement ratio (1.5%)	S-H-1.5 (corroded control)	–	–	–	–
	S-1.5	1	1.2	50	skewed FRP sheets
	O-1.5	1	1.2	50	orthogonal FRP sheets
	O-R-1.5	1	1.2	0	orthogonal-radial FRP sheets
	S-R-1.5	1	1.2	0	skewed-radial FRP sheets
Number of layers	S-H-C1	1	1.2	50	Skewed FRP sheets
	S-H-C2	2	1.2	50	Skewed FRP sheets
	S-H-C3	3	1.2	50	Skewed FRP sheets
	S-H-C4	4	1.2	50	Skewed FRP sheets
	S-H-C5	5	1.2	0	Skewed FRP sheets
FRP thickness	S-H-C1-0.2	1	0.2	50	Skewed FRP sheets
	S-H-C1-0.6	1	0.6	50	Skewed FRP sheets
	S-H-C1-1.2	1	1.2	50	Skewed FRP sheets
	S-H-C1-1.6	1	1.6	50	Skewed FRP sheets
	S-H-C1-2	1	2	50	Skewed FRP sheets
FRP location from column	S-H-C1-0	1	1.2	0	Skewed FRP sheets
	S-H-C1-15	1	1.2	15	Skewed FRP sheets
	S-H-C1-30	1	1.2	30	Skewed FRP sheets
	S-H-C1-50	1	1.2	50	Skewed FRP sheets
Strengthening schemes	S-H-GC	2	1.2	50	Skewed FRP sheets
	S-H-CG	2	1.2	50	Skewed FRP sheets
	S-H-CC	2	1.2	50	Skewed FRP sheets
	S-H-GG	2	1.2	50	Skewed FRP sheets
	S-H-GGG	3	1.2	50	Skewed FRP sheets
	S-H-GCG	3	1.2	50	Skewed FRP sheets
	S-H-GGC	3	1.2	50	Skewed FRP sheets
	S-H-GCC	3	1.2	50	Skewed FRP sheets
	S-H-CCC	3	1.2	50	Skewed FRP sheets
	S-H-CGC	3	1.2	50	Skewed FRP sheets
	S-H-CCG	3	1.2	50	Skewed FRP sheets
	S-H-CGG	3	1.2	50	Skewed FRP sheets

#### Strengthening configurations with different tensile reinforcement ratio

**Fig. 11:**
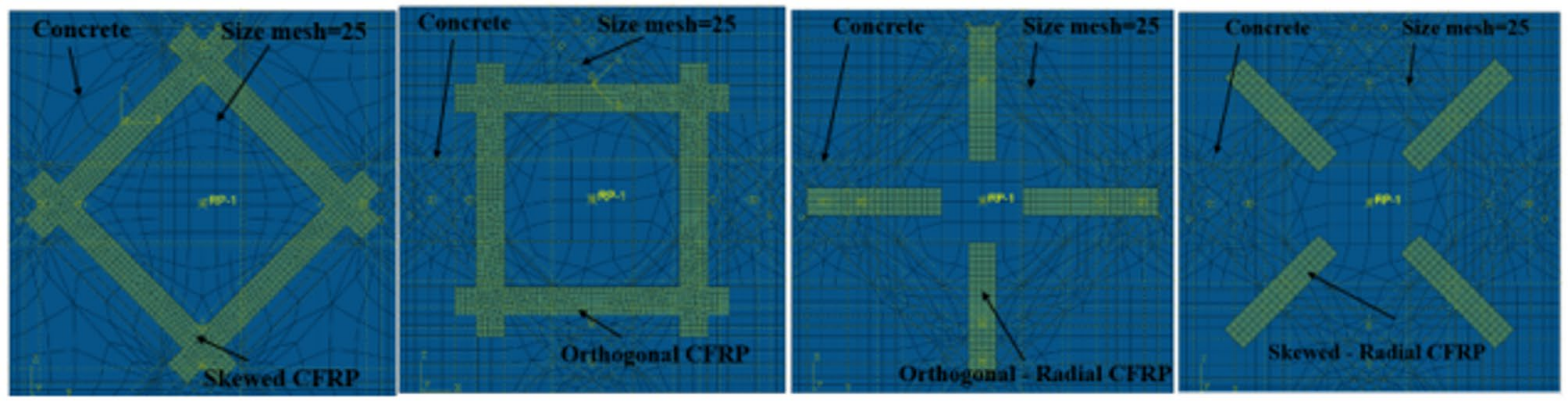
Finite element model.

**Fig. 12:**
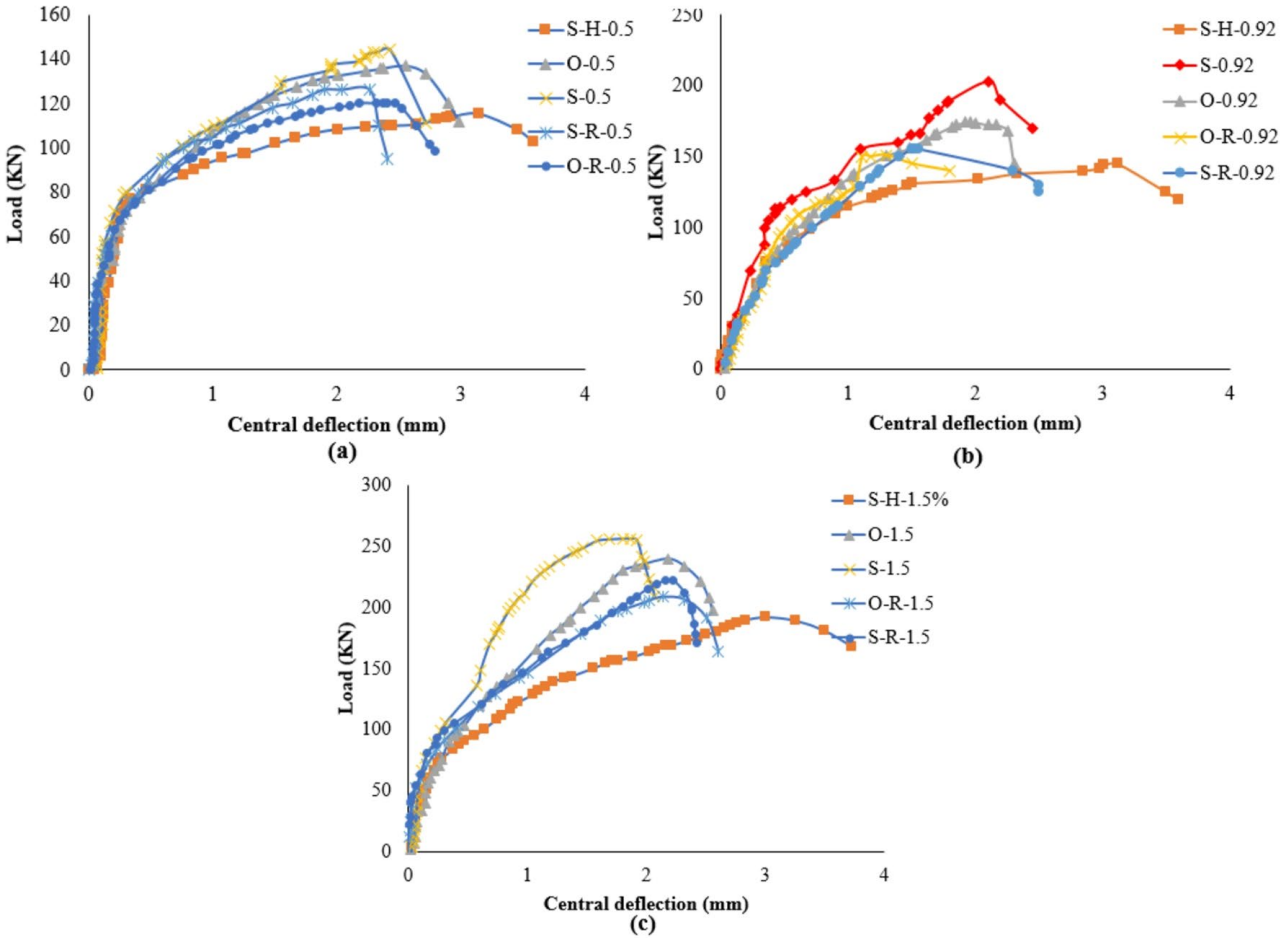
Load–deflection curves for various strengthening configurations at different tensile reinforcement ratios: (a) low tensile reinforcement ratio; (b) moderate tensile reinforcement ratio; (c) high tensile reinforcement ratio.

Figure 11 illustrates the four CFRP configurations analyzed. Load-deflection curves for the slabs are shown in Fig. 12. Results show that skewed strip configurations had higher PS capacity than other setups. Failure modes varied with reinforcement ratio; higher reinforcement led to punching failure, while lower and moderate reinforcement resulted in flexure or flexure punching. Strengthening with skewed and orthogonal strips changed SCJs from flexure to punching failure, as listed in Table S5 (supplementary materials).

#### Number of layers

**Fig. 13:**
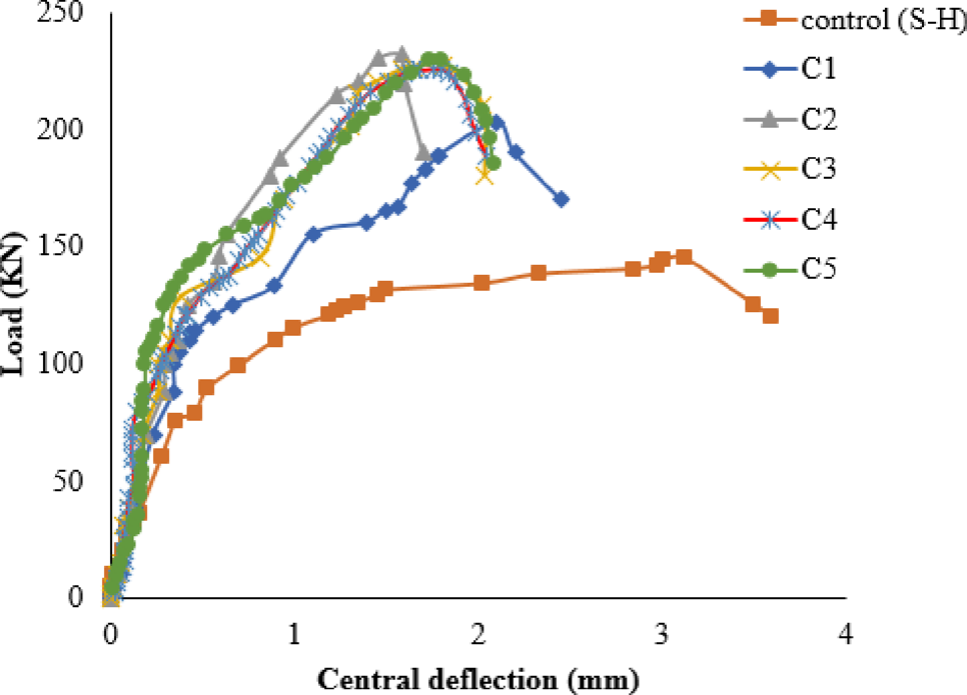
Load-deflection curves for various numbers of layers.

Figure 13 shows load-deflection curves for specimen S-H with varying numbers of CFRP layers. Using one CFRP layer increases PS capacity by 40% and reduces ultimate deflection by 28.5% compared to the S-H specimen. Adding two CFRP layers increases ultimate PS capacity by 66.8% and reduces ultimate deflection by 49.3% compared to the S-H specimen. However, increasing the number of CFRP layers from 3 to 5 only slightly increases PS capacity from 57% to 60% compared to the S-H specimen. The results in Table S5 (supplementary materials) show that adding up to two layers of FRP sheets leads to debonding of the FRP sheets.

#### FRP thickness

**Fig. 14:**
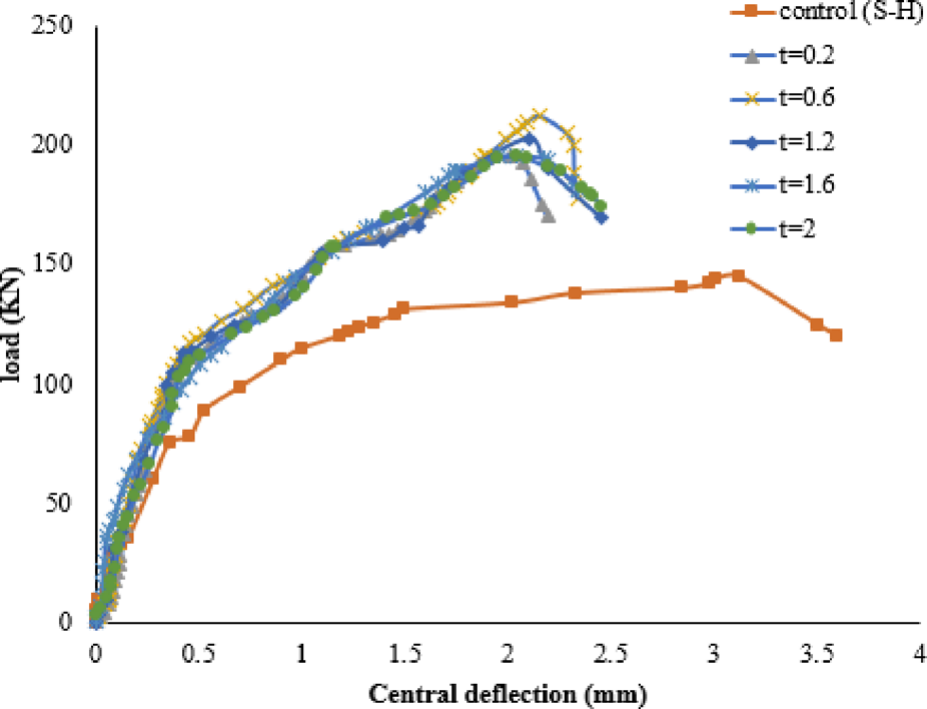
Load-deflection curves for various layer thicknesses.

This part investigates the impact of varying the thickness of FRP sheets on strengthening RC SCJS, considering its effect on load capacities and deflections. Figure 14 shows load-deflection curves for specimens S-H with different CFRP thicknesses. Increasing the CFRP thickness enhances the PS capacity due to additional concrete confinement, resulting in improved resistance to shear stresses. Specifically, increasing the CFRP thickness from 0.2 mm to 2 mm raises the PS capacity from 145 kN to 210 kN compared to specimen S-H. However, using different thicknesses of CFRP layers does not significantly affect the PS capacities, with the optimal thickness ranging between 0.6 mm and 1.2 mm. Findings from Table S5 (supplementary materials) suggest that using up to 1.2 mm FRP thickness may lead to FRP debonding from the concrete surface.

#### FRP location from the column face

**Fig. 15:**
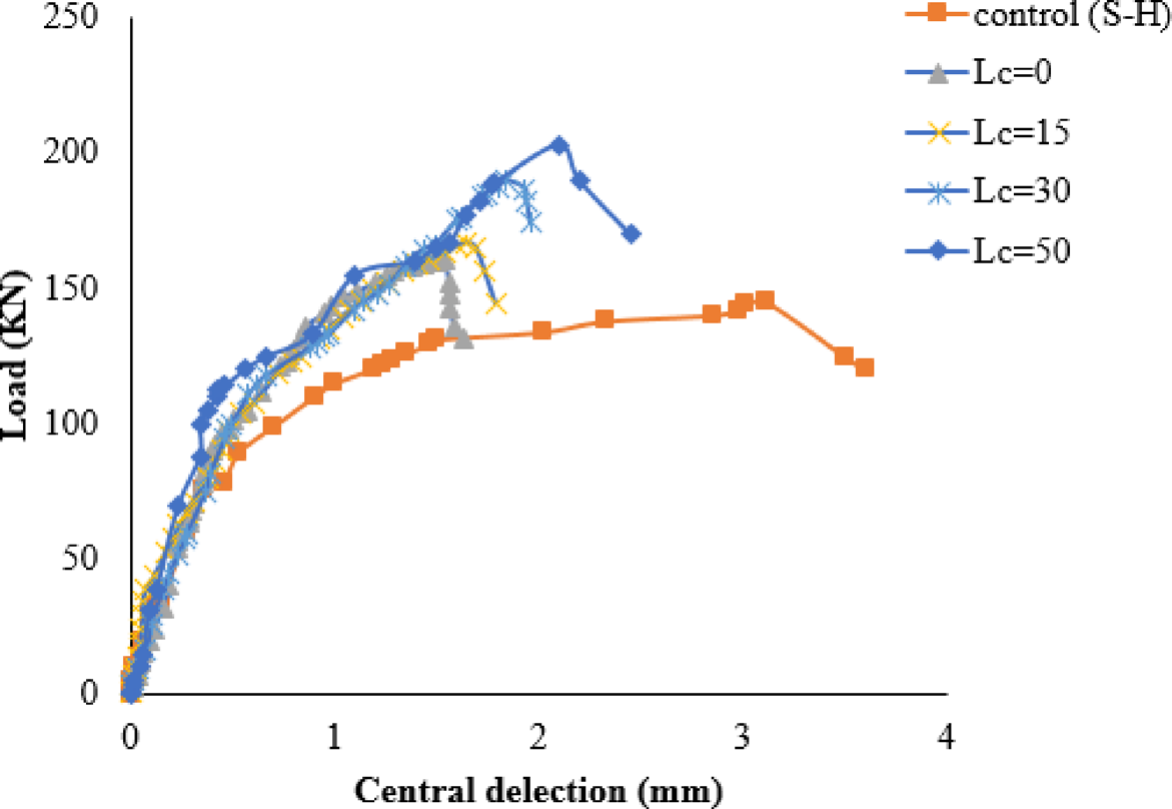
Load-deflection curves for various FRP locations from the column face.

Figure 15 depicts the load-deflection curves of different locations of the CFRP strips from the column face. It can be observed that the PS capacity increases as the distance between the strips and the column face increases. As can be seen from Fig. 15, increasing the location of the strips from the column face from 0.0 mm to 50 mm increases the PS capacity from 145kN to 203kN, respectively, compared with specimen S-H. Based on these results, it can be inferred that the most successful configuration was the skewed strip arrangement with a column face offset by 50 mm. These results are consistent with another research^24,74^.The results demonstrate that the failure mode remains consistent when the distance of the strips is increased from 0.0 mm to 50 mm away from the column face, as presented in Table S5 (supplementary materials).

#### Strengthening schemes

**Fig. 16:**
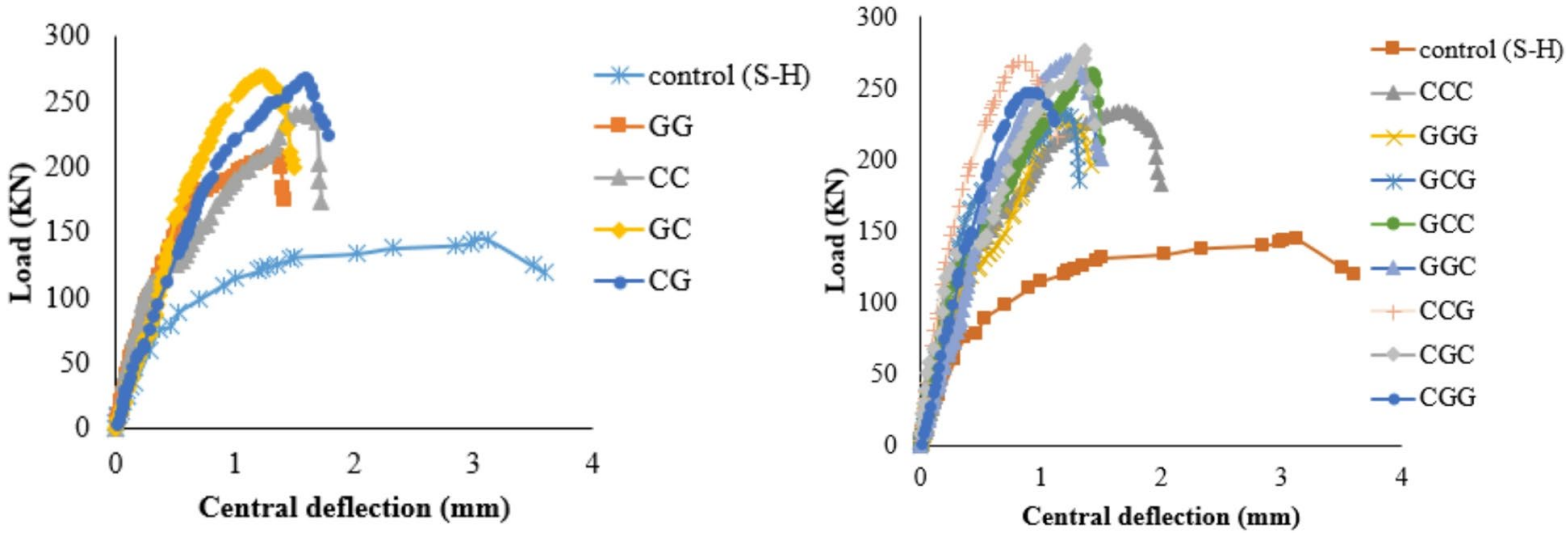
Load-deflection curves for various strengthening schemes.

Twelve distinct strengthening schemes with the same number of layers were simulated using FE to examine the impact of the lay-up order of glass and carbon layers, as shown in Table 5. For the specimens strengthened using 2 layers, the PS capacity of specimen S-H-GC exceeds specimen S-H-GG and S-H-CC by 28.2% and 11.1%,respectively, as shown in Fig. 16. It is noted that the PS capacity of specimens S-H-GC compared with S-H-CG still constant, but the ultimate deflection increases by 29% compared with the specimen S-H-GC. This can be attributed to the fact that CFRP typically exhibits lower slip behavior compared to GFRP due to its higher stiffness and bond strength with the adhesive and concrete. As a result, when CFRP is placed adjacent to the concrete surface, it tends to provide better confinement and restraint against lateral movement, leading to reduced deflection. The combination of different FRP materials can lead to improved load transfer and better overall performance, resulting in enhanced deflection capacity compared to using only one type of FRP, as listed in Table S5 (supplementary materials).

### Corrosion effect

In this part, the effect of corrosion on the strengthening efficiency is studied by changing the corrosion ratios and corroded areas. Based on previous parametric study results, it is found that the optimal strengthening technique was the skewed strip arrangement with a column face offset by 50 mm and two layers with a thickness of 0.6 for a 15% corrosion ratio. To reduce the number of models, optimal strengthening techniques with different ratios of corrosion, and corroded area, were studied.

#### Impact of corrosion ratio

**Fig. 17:**
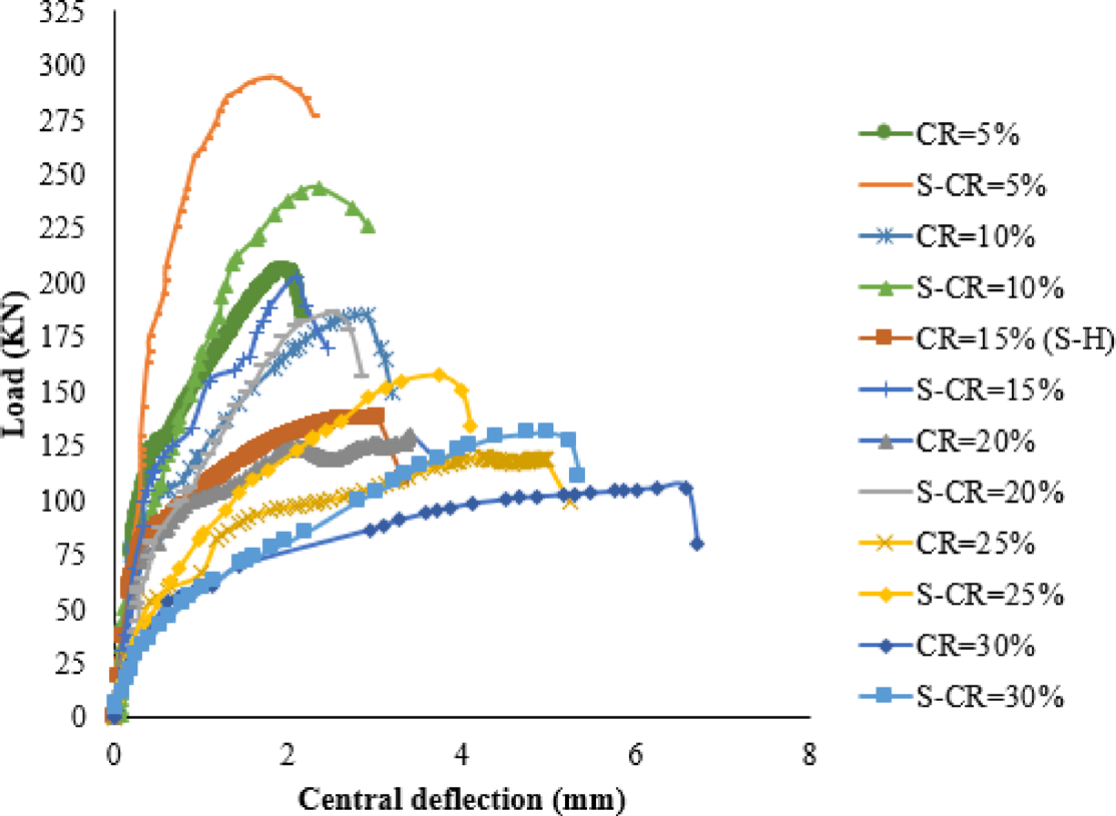
Load-deflection curves for different corrosion ratios.

In this part, six corroded joints with corrosion ratios of 5%, 10%, 15%, 20%, 25%, and 30%areinvestigated under optimal strengthening techniques, as tabulated in Table S6 (supplementary materials). As shown in Fig. 17, increasing the CR decreases the PS capacity and raises the ultimate deflection. The results show that as the CR increases from 5% to 30%, the strengthening efficiency decreases from 50.77% to24.76%. It is noted that as the CR increases above15%, the failure mode changes to punching/debonding failure, as tabulated in Table S7 (supplementary materials).

#### Impact of the corroded area

**Fig. 18:**
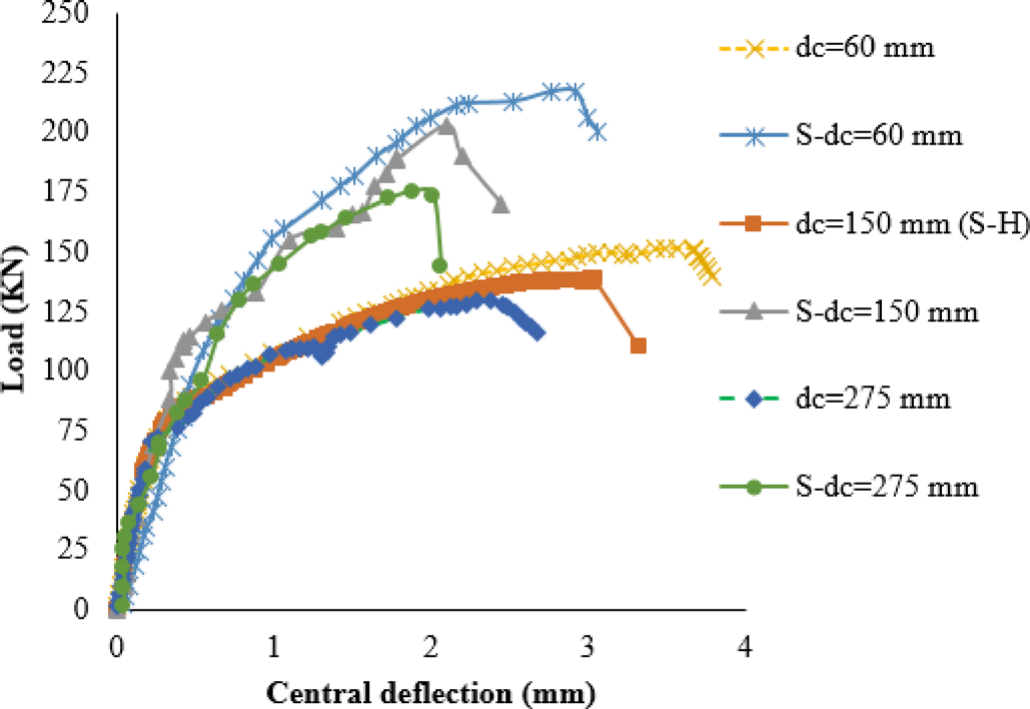
Load-deflection curves for different corrosion areas.

The impact of the corroded area is examined by considering three different distances (dc) from the column face, as specified in Table S8 (supplementary materials). Two values of dc (60 mm and 150 mm) are investigated to represent local corrosion scenarios, while a third value of dc (275 mm) is utilized to simulate global corrosion. The findings reveal that as the corroded zone’s area increases, the efficiency of strengthening experiences a slight decrease, as presented in Fig. 18. Additionally, it is observed that despite the increase in the corroded area, the failure mode remains consistent, as summarized in Table S9 (supplementary materials).

### Analysis of full-scale model

**Fig. 19:**
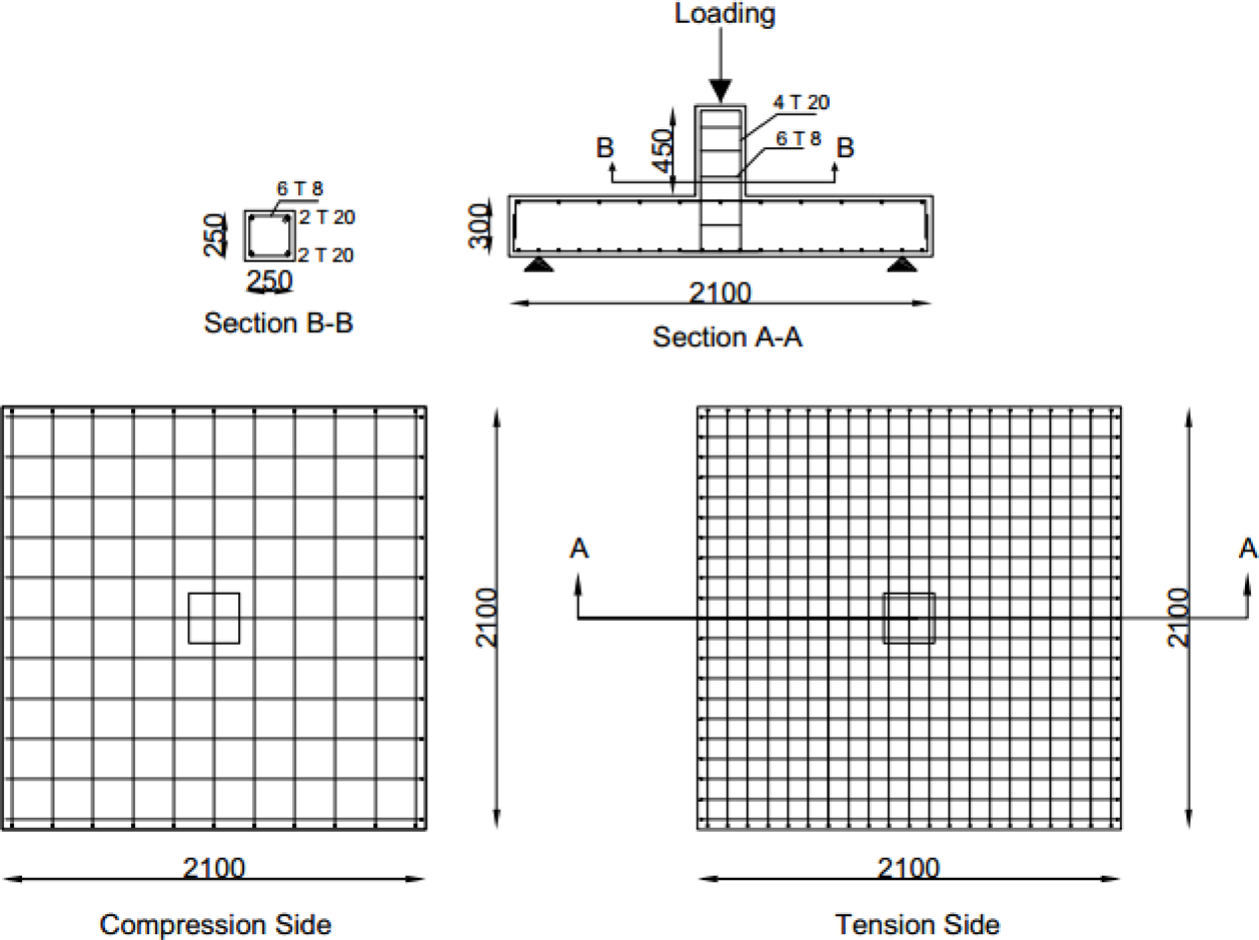
Dimensions and details of a full-scale model.

**Fig. 20:**
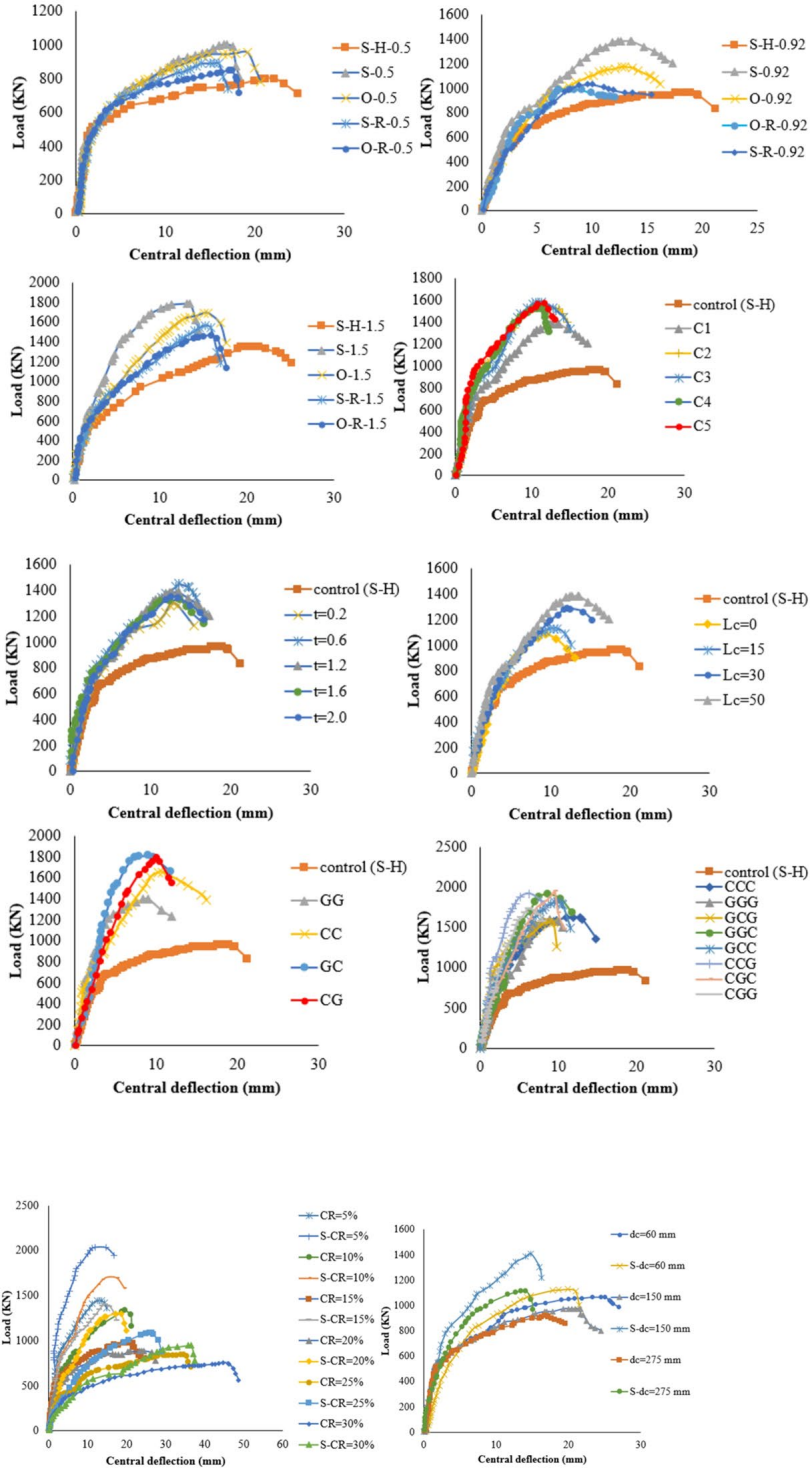
Load-deflection curves of full-scale models.

In this study, several full-scale models were simulated using ABAQUS to explore the impact of size effect on the PS capacity of the RC SCJS, with all previously evaluated parameters considered. The full-scale reference model’s dimensions and details are shown in Fig. 19, with the same steel and concrete properties as the scaled model. The full-scale model of the slab maintained a slab reinforcement ratio of 0.92%.The load-deflection curves of the full-scale models are presented in Fig. 20. Overall, the changes in load-deflection curves of the full-scale models correspond to those of the scaled models for the various parameters.

## Discussion

The numerical and experimental results are now discussed in detail in the following sections. Based on numerical and experimental results, it can be inferred that the most successful configuration for CR 15% was the skewed strip arrangement with a column face offset by 50 mm and two layers with a thickness of 0.6 mm. The parametric investigation demonstrated that the factors under consideration exhibit varying effects on the behavior of corroded RC SCJs. These results are partially consistent with previous observations; however, the present study provides additional quantitative insight into the interaction between corrosion severity, hybrid FRP configurations, and PS response, which has not been previously examined. While the proposed hybrid thin-ply FRP configurations showed superior performance relative to the tested CFRP and GFRP systems, the findings should not be interpreted as universally superior. The effectiveness is directly linked to bond conditions, corrosion severity, and the specific geometry parameters examined in this study.

The results of this study are consistent with previous research on the punching shear behavior and strengthening of RC slab–column connections. Qian et al.^1^ reported that corrosion significantly reduces punching shear capacity due to the loss of aggregate interlock and dowel action, which aligns with the 33% reduction observed in the present study. Similarly, Silva et al.^75^ found that externally bonded CFRP strips can partially restore the lost capacity of deteriorated slab–column joints, although debonding remains a common failure mode also observed in the strengthened specimens here. Soudki et al.^24^ and Abdulrahman et al.^72^ further noted that FRP strip orientation strongly influences the effectiveness of strengthening, with inclined or skewed configurations providing better confinement against radial punching cracks. This agrees with our finding that the skewed hybrid FRP arrangement with a 50 mm offset achieved the highest strengthening efficiency. Additionally, studies on hybrid FRP systems have demonstrated superior stiffness recovery and enhanced energy absorption compared to single FRP types, supporting the improved performance of thin-ply glass/carbon hybrids observed in this research. Overall, the outcomes of this study not only confirm trends reported in the literature but also extend the current understanding by incorporating corrosion effects, hybrid FRP behavior, and detailed FE-based parametric investigation.

### Strengthening configurations

The findings indicate that an increase in the reinforcement ratio notably enhances the PS capacity. Existing literature suggests that in slabs with high reinforcement ratios, the flexure capacity may exceed the PS capacity, leading to brittle PS failure. On the other hand, slabs with low reinforcement ratios may undergo flexure failure with higher deformation capacity, as the SCJ flexure capacity is lower compared to the PS capacity^21,76–79^. In the presence of corrosion, the failure mode is affected not only by the reinforcement ratio but also by concrete deterioration from stresses induced by corrosion products at the steel-concrete interface. The enhanced PS capacity with increased reinforcement ratio is attributed to the greater dowel forces developed in the flexural reinforcement, resisting inclined shear cracks. These findings agree with other research^2,21^.The results demonstrate that the skewed strip configurations resulted in a higher PS capacity compared to the other configurations. This is because the strips of the skewed strengthening pattern were oriented differently than the inner reinforcement, which was oriented orthogonally, radially orthogonally, or radially skewed. Considering that PS cracks are distributed in a radial pattern, the skewed CFRP strips with orthogonal internal reinforcement are more effective at preventing the expansion of these cracks. Also, the skewed strip arrangement redistributes the shear stresses in a way that the critical shear regions are subjected to lower stress levels. This helps in delaying the initiation of PS failure and allows the structure to accommodate higher loads before reaching the ultimate failure state. These results are consistent with other research^24,74^. Utilizing FRPs on the tension surface of RC slabs with a low tensile reinforcement ratio proves to be an effective approach for enhancing load capacity. Nevertheless, it may lead to decreased slab deflection and ductility. The higher load capacity observed in FRP-strengthened RC SCJs with a low tensile reinforcement ratio can be attributed to the overall increase in tensile reinforcement within the slab, aligning with similar findings in previous studies^80^.

Unlike previous studies, the current work quantifies the performance difference between FRP orientations under corrosion-induced degradation and demonstrates that the skewed hybrid configuration provides up to 76.55% capacity enhancement, an insight not reported in prior slab–column strengthening research.

### FRP thickness

The study highlights the importance of careful consideration when choosing the thickness and quantity of FRPs for strengthening corroded RC SCJs to meet the specific requirements of the joint. The findings suggest that increasing the number of FRP layers could improve the joint’s PS capacity if it does not cause FRP debonding. However, it is essential to be aware that this approach may also result in reduced joint ductility. Therefore, a well-balanced approach is essential to achieve effective strengthening while maintaining sufficient ductile behavior in the joints.

The findings show that specimens with thicknesses of 0.6 mm and 1.2 mm exhibited the highest PS capacity before failure. While increasing the FRP thickness from 0.3 mm to 0.6 mm or increasing from one to two layers resulted in noticeable gains in PS capacity, further increases beyond two layers or beyond approximately 0.6–1.2 mm produced only marginal improvements (< 5%) because premature debonding governed the response. Thus, the strengthening effect saturates once the effective bond and crack-bridging capacity of the FRP system is reached. However, specimens with thicknesses of 1.6 mm and 2.0 mm experienced failure due to the debonding of the strengthening material. Thin fiber layer attains stable and gradual damage process, however a single and sudden occurred in thick layer. Another justification may be attributed due to the effects of corrosion or high shear inter-laminar stress. Corrosion can weaken the bond between steel reinforcement and concrete, affecting the effectiveness of strengthening measures. The likelihood of FRP debonding increases with higher initial tensile reinforcement ratios, as observed in other research^81,82^.

During the strengthening process and subsequent testing, several differences in failure mechanisms were observed across the various FRP arrangements. Specimens strengthened with orthogonal or radial FRP strips frequently experienced premature debonding, particularly when multiple layers were used. This behavior can be attributed to high interfacial shear stresses and limited compatibility between the FRP orientation and the predominant radial punching shear cracks. In contrast, the skewed FRP configuration demonstrated superior performance, as the strip orientation intersected the radial crack paths more effectively, resulting in enhanced crack bridging and delayed crack propagation. Additionally, hybrid glass–carbon FRP systems exhibited improved behavior compared to single-material FRP due to the combined benefits of GFRP’s higher strain capacity and CFRP’s stiffness, which produced a more balanced stress distribution and reduced stress concentrations at the adhesive interface. These factors explain why the skewed hybrid FRP arrangements consistently achieved higher punching shear capacity, greater stiffness, and more stable load–deflection behavior compared to the other strengthening configurations.

This study identifies 0.6 mm as the performance threshold beyond which increased thickness no longer enhances PS capacity due to premature debonding providing a clear quantitative design limit not addressed in earlier literature.

### FRP location from the column face

The most successful configuration was the skewed strip arrangement with a 50 mm column face offset. This larger offset provides a longer effective lever arm for the PS force to act on the slab, leading to a more efficient load transfer and higher resistance against PS failure. Additionally, the offset configuration allows for a gradual distribution of the PS force from the column face to the slab’s perimeter, reducing stress concentrations and ensuring better utilization of the slab’s capacity to resist punching. These findings align with other research^24,74^.

### Corrosion

The study reveals that increasing the size of the corroded zone negatively impacts the PS capacity of the specimens. This reduction is primarily attributed to the overall weakening of the specimens as the corroded area expands. However, it is worth noting that the decline in PS capacity is not directly proportional to the extent of the corrosion zone. Previous research has indicated that only the reinforcement within 1.5 times the distance from the column face effectively resists the PS force^83–86^. The current study’s findings are in agreement with this conclusion, as the PS capacity decreases by only 11% when the corrosion distance (dc) increases from 150 to 275 mm (from 1.5 times to 3.2 times the distance, d_o_). Similarly, a decrease in dc from 150 to 60 mm leads to a mere 4.13% improvement in the PS capacity. The results also demonstrate that as the corrosion ratio increases from 5% to 30%, the efficiency of strengthening decreases from 50.77% to 24.76%. Notably, the failure mode remains consistent despite the increase in the area of the corroded zone.Several factors contribute to the decrease in strengthening efficiency with an increasing corroded zone area. Corrosion of the rebar may weaken the bond between the FRP and the concrete surface, leading to reduced load-carrying capacity. Additionally, the presence of corrosion-induced cracks and damage in the concrete can affect the overall structural integrity, limiting the effectiveness of the FRP in reinforcing the SCJ.

### Ductility

The conventional definition of ductility, as stated in reference^87^, is the ratio of ultimate deflection to the deflection at the first yielding of the flexural rebars. However, this definition is not suitable for corroded structures due to practical challenges in measuring rebar yield. To address this, the ductility index term was introduced in reference^88^, defined as the ratio of the deflection at the ultimate load in the corroded element to that of the non-corroded one. Test results reveal that increasing the CR raises the ultimate deflection, resulting in an increase in the ductility index. Higher ductility is observed in pure flexural and flexural punching failures than in brittle punching failures, which occur with comparatively high tensile reinforcements. This is because the addition of FRP composites increases the stiffness of the structure, reducing its ability to undergo plastic deformation before failure. Furthermore, the ductility index values generally decrease with an increasing number of FRP layers. As the number of FRP layers increases, the stiffness of the structure also increases, limiting its ability to undergo significant deformation and elongation before failure, resulting in reduced ductility.

### Size effect

Span-to-depth (L/d) effect or the size effect is one of the outstanding aspects of fracture mechanics. Lovrovich and McLean^89^ in 1990 concluded that PS capacity significantly increases as the span-to-depth ratio decreases below 6. In the present experimental program, L/d = 7.05 for all specimens.In this study, the changes in load-deflection curves of the full-scale models correspond to those of the scaled models for the various parameters. These results are consistent with other research^2,89,90^.

### Assumptions, study limitations, and future research

Several assumptions were adopted in the experimental and numerical program to enable a controlled evaluation of punching shear behavior under corrosion and FRP strengthening. The corrosion process was assumed to be uniform along the reinforcement length, while in real structures corrosion may be localized or non-uniform due to variable exposure conditions. The adopted bond–slip relationships and cohesive interface parameters were based on established models and calibrated against experimental observations; however, potential variations in adhesive quality, surface preparation, or construction workmanship may influence actual bond performance. In the FE simulations, a perfect FRP–concrete interface prior to damage initiation was assumed, and environmental effects such as temperature, moisture, freeze–thaw cycles, and time-dependent concrete shrinkage or steel creep were not considered.

The strengthening layouts were chosen based on practical constructability, yet only a limited range of hybrid FRP widths, thicknesses, and orientations were examined. The corrosion ratios analyzed (5–30%) represent moderate to severe deterioration levels; therefore, the behavior at very early corrosion stages (< 5%) or highly advanced deterioration (> 30%) remains unverified. In addition, while the numerical model showed strong agreement with test data, it does not capture progressive bond degradation, FRP aging, or durability under cyclic environmental exposure, which may affect long-term strengthening performance.

Future research should investigate hybrid FRP systems under cyclic, fatigue, and dynamic loading, particularly for applications in seismic regions or structures subjected to repetitive service loads. Broader experimental and numerical parametric studies incorporating non-uniform corrosion patterns, different slab thicknesses, reinforcement ratios, and column geometries would enhance the generality of the findings. Additional work on anchorage systems, mechanical fasteners, and anti-debonding techniques is recommended to mitigate premature debonding and extend the effective strengthening threshold beyond the 0.6 mm thickness identified here. Incorporating probabilistic corrosion models and long-term environmental deterioration mechanisms could support the development of more robust and reliable design recommendations for corroded slab–column joints strengthened with thin-ply hybrid FRP.

## Conclusions

Based on the experimental results, validated numerical modeling, and the conducted parametric investigation, the following conclusions are drawn within the scope of the parameters investigated in this study:

1. Corrosion significantly alters structural response, reducing punching shear (PS) capacity and shifting failure from ductile flexural behavior toward more brittle flexural–punching shear modes. This transition was quantified for thin-ply FRP–strengthened specimens and shown to directly influence the level of achievable strengthening.
2. The observed reduction in stiffness and PS capacity with higher corrosion ratios is linked to degradation of aggregate interlock, dowel action, and steel–concrete bond. The measured trends and corresponding changes in failure mode provide clearer mechanistic evidence of corrosion–strengthening interaction than previously reported.
3. Hybrid glass–carbon FRP exhibited the highest strengthening efficiency among the tested configurations, with capacity increases of approximately 57–77% relative to corroded controls. These outcomes reflect the combined benefits of CFRP stiffness and GFRP strain capacity; however, this behavior is specific to the investigated slab geometry, corrosion level, and material configuration.
4. The developed ABAQUS modeling framework, which incorporates corrosion-induced material degradation and cohesive bond–slip behavior, reproduced load–deflection responses, cracking patterns, and debonding initiation with strong agreement to experiments. This supports the use of the model as a tool for assessing deteriorated slab–column joints under similar conditions.
5. Full-scale numerical models indicated that the strengthening trends found in the scaled specimens remained consistent for L/d > 6, suggesting limited size dependency within common flat-slab proportions. Extrapolation to significantly different thicknesses or reinforcement layouts may require further validation.
6. An optimal configuration was identified for the studied corrosion level (≈ 15%): two hybrid layers (0.6 mm total thickness) arranged in a skewed layout with a 50 mm offset from the column face. This arrangement most effectively intercepted radial punching cracks and delayed debonding, providing practical guidance for similar retrofit scenarios.
7. Increasing FRP layers beyond two or thickness beyond approximately 0.6 mm did not produce proportional capacity gains due to premature debonding. These findings indicate a performance threshold where bond capacity governs the response more than increased material stiffness.
8. Strengthening efficiency decreased with corrosion severity, falling from ≈ 51% at 5% corrosion to ≈ 25% at 30% corrosion. The data further show that increased corroded area has limited influence unless deterioration extends within ≈ 1.5d from the column face, where reinforcement directly contributes to punching resistance.
9. Failure of strengthened specimens remained governed by punching shear or punching accompanied by FRP debonding. Once debonding initiated, further load gains were limited, indicating that bond performance currently controls the upper bound of recoverable strength for this system.
10. The addition of FRP layers increased stiffness but reduced deformation capacity, leading to a lower ductility index. This emphasizes a strength–ductility trade-off that should be considered in retrofit design, particularly for structures requiring deformation compatibility under seismic or cyclic demands.

## Supplementary Information

Below is the link to the electronic supplementary material.

Supplementary Material 1

## Data Availability

Availability of data and materials All data generated or analyzed during this study are included in this published article.
