# Supplementary material for: Strengthening of corroded RC slab–column joints using thin-ply hybrid FRP under punching shear
Source: Sci Rep. 2026 Feb 14;16:6526. doi: 10.1038/s41598-026-36610-2 (PMC12909307; doi:10.1038/s41598-026-36610-2)
Supplement: Supplementary file 1 — Supplementary Material 1 [file 41598_2026_36610_MOESM1_ESM.docx]

**Supplementary File**

**Table S1:** Plastic damage parameters used for modeling.

| Parameter | Value |
| --- | --- |
| Poisson’s ratio of concrete | 0.2 |
| Poisson’s ratio of steel | 0.3 |
| elastic modulus f concrete | 30000 |
| elastic modulus of steel | 200000 |
| Dilation angle (ψ) | 30° |
| Eccentricity (ε) | 0.1 |
| ratio of initial equi biaxial compressive yield stress to initial uniaxial compressive yield stress (f_b0_/f_c0)_ | 1.16 |
| the ratio of the second stress invariant on the tensile meridian (K_c)_ | 0.667 |
| viscosity parameter (µ) | 0.0005 |

**Table S2:** Comparison of yield stress and ultimate stress between experimental and mathematical equations.

| specimen code | Theoretical | | Fy | Fu |
| --- | --- | --- | --- | --- |
|  | Fy | Fu | (Exp/Theoretical) | (Exp/Theoretical) |
| S1-L | 453.99 | 585.31 | 0.99 | 1.00 |
| S1-M | 423.98 | 546.62 | 0.99 | 0.99 |
| S1-H | 393.97 | 507.93 | 0.94 | 0.96 |

**Table S3:** Comparison of bond strength between experimental and mathematical equations.

| specimen code | Al-Sakkaf, et al[1] | |
| --- | --- | --- |
|  | $\tau_{\max}^{R}$ (Exp/Theoretical) | $S_{max}$ (Exp/Theoretical) |
| S1-L | 1.12 | 1.03 |
| S1-M | 1.04 | 1.06 |
| S1-H | 0.98 | 0.98 |

**Table S4:** Design codes

| Design methods | Punching shear strength (Mpa) | Critical section $b_{o}$ |
| --- | --- | --- |
| ACI 318–19 ^52^ | Minimum of  $V_{c= \frac{1}{12} (1+\frac{4}{\beta})\lambda s\lambda\sqrt{f_{c}^{-}}b_{o}d}$  $V_{c= \frac{1}{12} (\frac{\alpha sd}{b_{o}}+2)\lambda s\lambda\sqrt{f_{c}^{-}}b_{o}d}$  $V_{c= \frac{1}{3}\lambda s\lambda\sqrt{f_{c}^{-}}b_{o}d}$ | 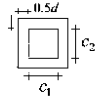 |
| CSA 23.3–14 ^53^ | Minimum of  $V_{c= 0.19 (1+\frac{2}{\beta})\lambda\varphi_{c}\sqrt{f_{c}^{-}}b_{o}d}$  $V_{c= (\frac{\alpha_{sd}}{b_{o}}+0.19)\lambda\varphi_{c}\sqrt{f_{c}^{-}}b_{o}d}$  $V_{c= 0.38\lambda\varphi_{c}\sqrt{f_{c}^{-}}b_{o}d}$ | 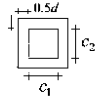 |
| Eurocode 2 ^55^ | Maximum of  $V_{Rd,c= 0.18k \left( 100\rho f_{c}^{-} \right)^{\frac{1}{3}}u1d}$  $V_{m}= 0.035k^{\frac{3}{2}\sqrt{f_{c}^{-}}}u1d$  Whare: $k=1+\sqrt{{200}/{d \leq2.0}}$ | 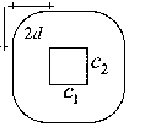 |
| BS 8110–97 ^54^ | $V_{c= 0.79 \left( 100\rho\right)^{\frac{1}{3}}\left( \frac{400}{d} \right)^{\frac{1}{4}}{(\frac{{f_{c}^{-}}/{0.78}}{25}}^{\frac{1}{3}}b_{o}d}$ | 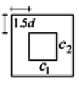 |

where**:**

| $C_{1,}C_{2}$ =size of rectangular column  $\beta$ is the column's long-to-short ratio  $\lambda s$ is size-related effects  $\lambda$ is the density of the concrete ($\lambda equal to 1 and 0.85$ for normal concrete and semi-lightweight concrete, respectively)  $\alpha s$is a factor based on column location ($\alpha s =40, 30, and 20$ for interior, edge, and corner columns, respectively)  $bo$ is the location of the critical section's perimeter  $d$ is the slab's effective flexural depth  $fc’$ is the compressive strength of concrete  $\varphi c$ is the concrete resistance factor  $\rho$ is the critical section's effective flexural reinforcement ratio  $k$ is the size effect coefficient  $u1 is the crucial shear perimeter positioned 2d from the column face; its corners are rounded.$ |
| --- |


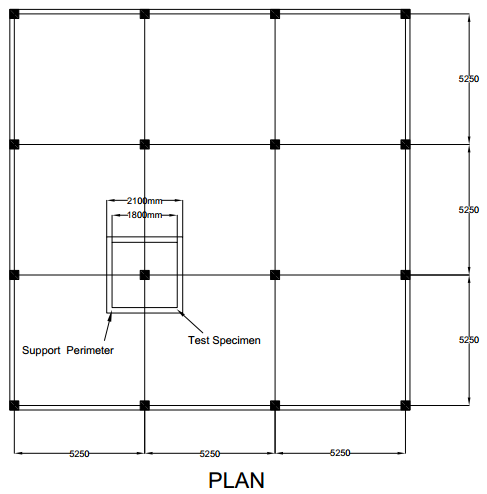


**Figure S1.** Plan view of Test Specimen Selection.


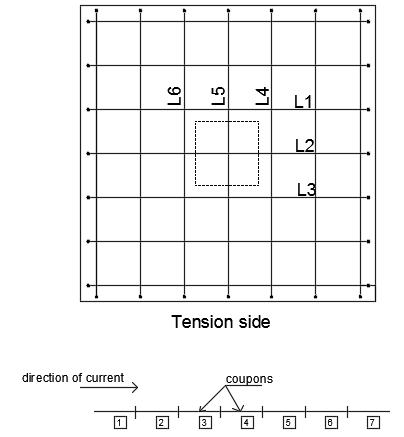


**Figure S2.** Arrangement of steel bars.


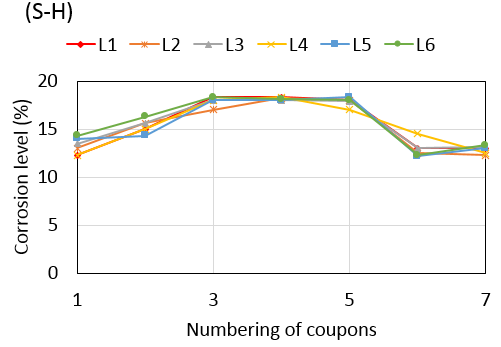

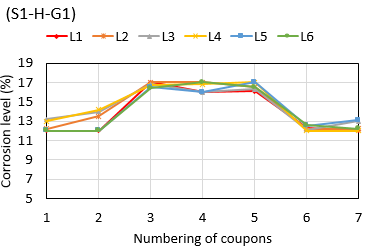


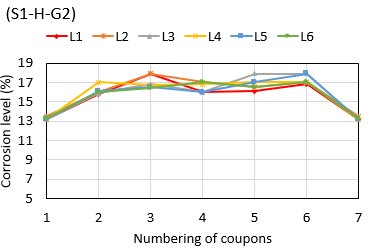

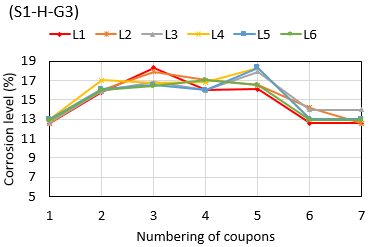


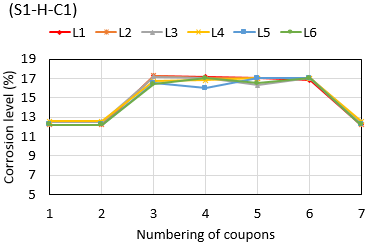

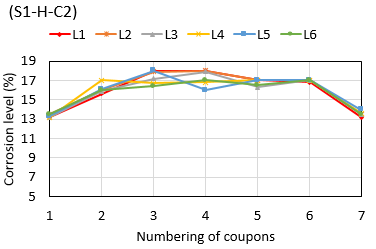


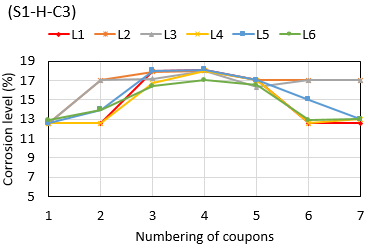

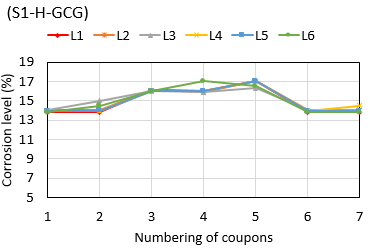


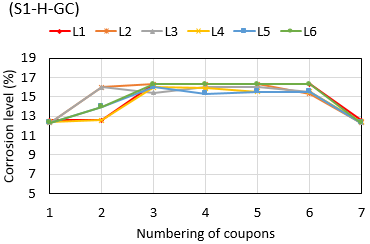

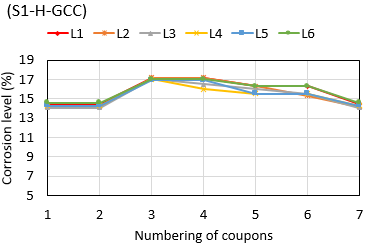


**Figure S3.** Distribution of corrosion level per bar length for each corroded specimens.


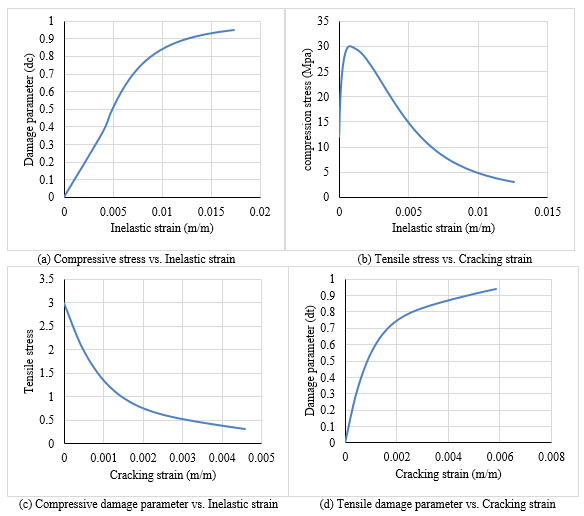


**Figure S4 .** Specification of the damage-plasticity model's parameters.


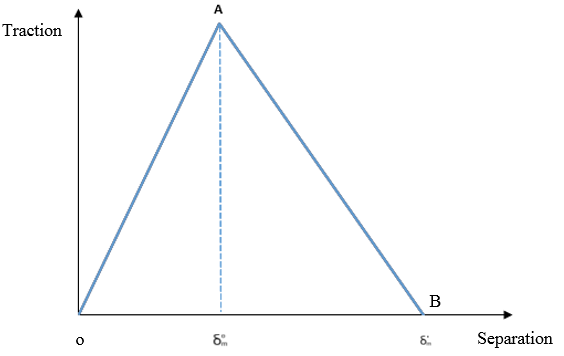


**Figure S5.** Traction-separation characteristic with linear damage evolution.


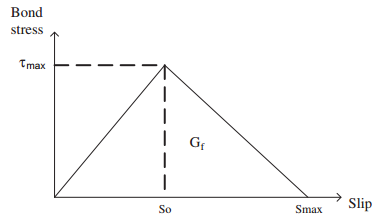


**Figure S6.** Cohesive model.


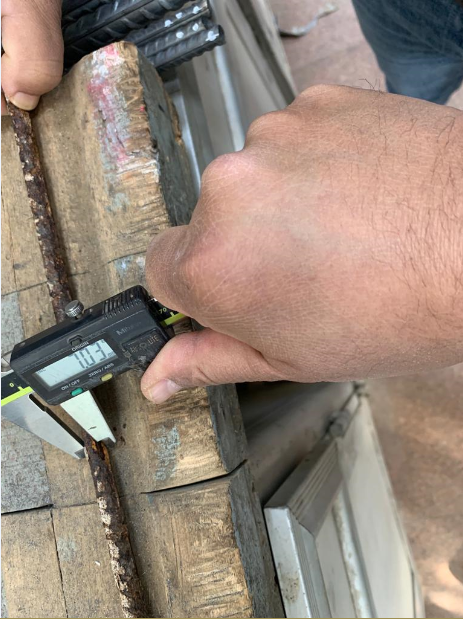

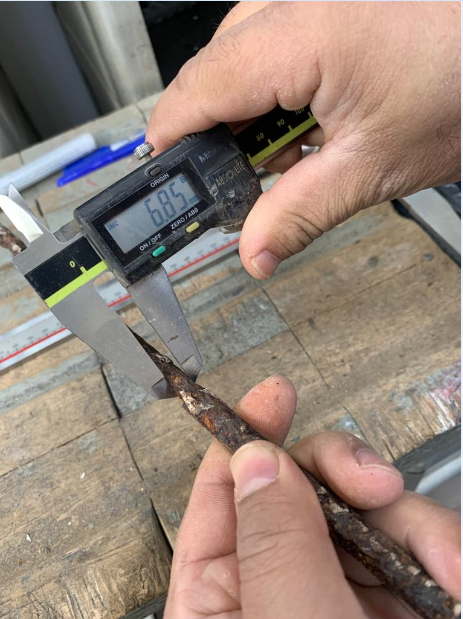

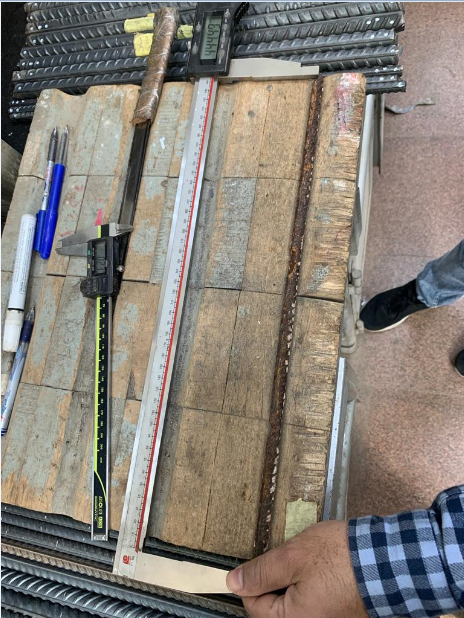


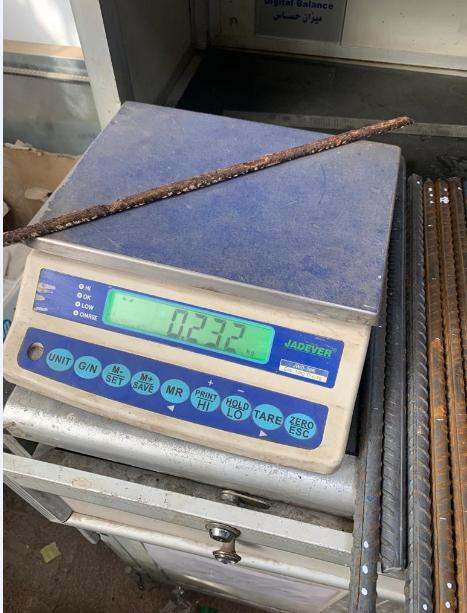

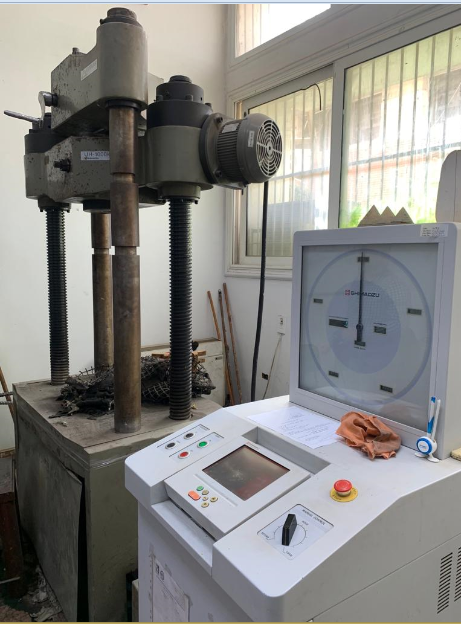

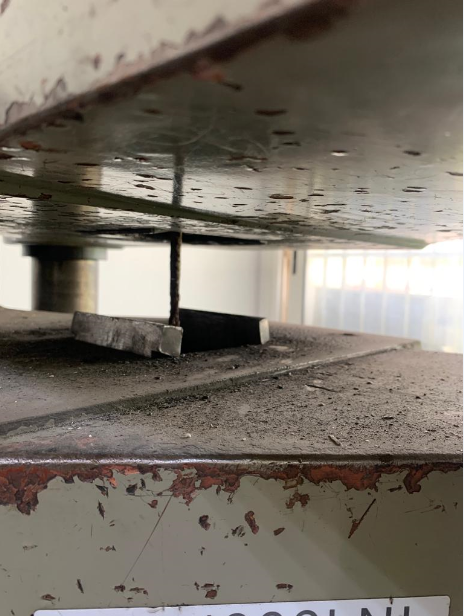


Experimental test to determine mechanical properties of corroded steel rebar.


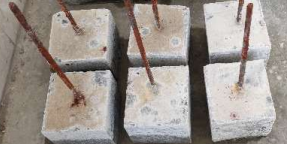


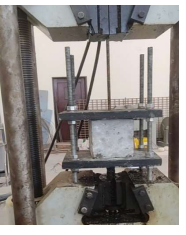

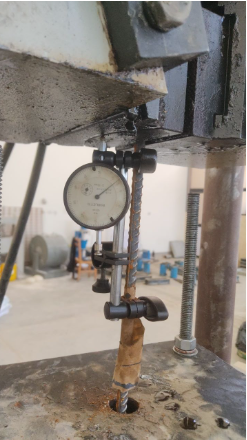

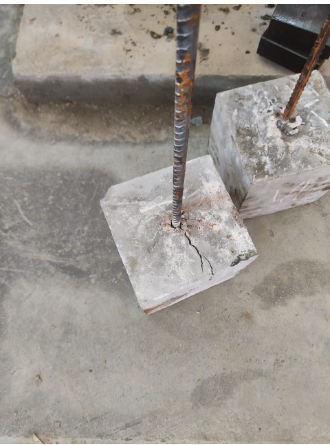


Pull-out Strength Device and Tested Specimens.

# Reference

1. Qian K, Li JS, Huang T, Weng YH, Deng XF. Punching shear strength of corroded reinforced concrete slab-column connections. *J Build Eng*. 2022;45(August 2021):103489. doi:10.1016/j.jobe.2021.103489

2. Silva MAL, Gamage JCPH, Fawzia S. Performance of slab-column connections of flat slabs strengthened with carbon fiber reinforced polymers. *Case Stud Constr Mater*. 2019;11:e00275. doi:10.1016/j.cscm.2019.e00275
